# An Untethered Ring-Shaped Miniature Robot with Axisymmetric Vibrations and Non-axisymmetrically Arranged Feet

**DOI:** 10.34133/research.1158

**Published:** 2026-02-19

**Authors:** Baoyi Liu, Jing Li, Yu Gao, Jinghan Guan, Boliang Xu, Jie Deng, Shijing Zhang, Yingxiang Liu

**Affiliations:** State Key Laboratory of Robotics and Systems, Harbin Institute of Technology, Harbin 150001, China.

## Abstract

Miniature robots inspired by nature are appealing, and the achievement of their rapidity has become uncomplicated after years of development. However, one of the critical challenges of miniature robots is achieving rapidity and agile motions under high load capacity. To address this challenge, this work proposes a unique tripodal piezoelectric robot, featuring axisymmetric vibration modes and non-axisymmetrically arranged feet. On the one hand, by selecting appropriate vibration modes and arranging the 3 feet at unequal spacing, multidimensional and unequal amplitude actuation trajectories at different feet are achieved, enabling the robot to achieve agile motions. On the other hand, strong load capacity is realized through a high-stiffness ring base. The selected vibration modes and different actuation trajectories at foot ends are validated through simulation analysis. A prototype of the robot is developed, and a miniature specialized power supply is designed and integrated into the robot. The wireless linear and rotational speeds reach 93 mm/s and 438°/s, respectively, and the load capacity reaches 200 g, while the resolution is 0.63 μm. Owing to its small size, high load capacity, high resolution, and agility, the robot demonstrates potential for micro-manipulation applications and motion capabilities in confined spaces.

## Introduction

In recent years, robotics has experienced rapid development. Centimeter-scale miniature robots (size: 1 to 10 cm, referred to as miniature robots in the following text) have become a research focus in the field of robotics due to their small size, lightweight, and agile motions, making them suitable for applications in micro-manipulation [1–5], biomedical engineering [6–10], multirobot collaborative operation [11–15], and rescue exploration [16–20]. Relevant studies have further demonstrated that the continuous advancement of these robots will broaden the boundaries of their practical applications in the mentioned fields. The rapidity of miniature robots has become relatively easy to achieve after years of development. However, a critical challenge is to balance agile motions and high load capacity while ensuring rapidity is achieved, which becomes even more severe as the size decreases. For instance, Hoover et al. [21] proposed a miniature robot “DynaRoACH” driven by an electromagnetic motor. The size of DynaRoACH is 10 cm; it can achieve a speed of 14 body length (BL)/s and can also achieve dynamic turning. Although this robot can achieve high-speed and agile motions, it has weak load-bearing capacity, and its structure is generally complex. With advancements in materials science, many smart materials have been applied in the field of miniature robots [22–28]. For example, Che et al. [29] designed a miniature robot driven by shape memory alloys, which can achieve various motions. Shape memory alloy-driven miniature robots exhibit good elasticity, but they have lower structural stiffness and generally respond slowly. The piezoelectric actuation technology based on piezoelectric ceramics offers advantages such as high precision, rapid response, and self-locking upon power failure [30–36]. Robots based on piezoelectric actuation (piezoelectric robots) have flexible structural design and are easy to miniaturize, providing possibilities for solving the key challenge of balancing rapidity, agile motions, and high load capacity [37].

To achieve agile motions, one usual solution is using multiple piezoelectric units in parallel to obtain multiple controllable driving force. For instance, Li et al. [38] proposed a miniature piezoelectric robot, which consists of 2 compact piezoelectric legs. It can achieve a maximum speed of 393.5 mm/s, and the load capacity reaches 100 g (4.8 times self-weight). Zhu et al. [39] introduced a piezoelectric robot composed of 2 compact units. It can realize agile motions, and the load capacity reaches 55 g (6 times self-weight). However, the connection parts of such a structure often utilize thin beam designs due to vibration isolation, resulting in lower stiffness. In our previous work, a quadruped ring-shaped piezoelectric robot with axisymmetric layout was proposed to balance the high speed, agile motion, and high load [40]; the results are 255 mm/s, 22 rad/s, and 200 g, respectively. However, the quadruped robot requires additional weight to ensure 3 feet can touch the ground and achieve stable motions, and its motions are restricted by external power supply.

This work designs a unique ring-shaped tripodal piezoelectric robot with non-axisymmetrically spaced feet. Strong load capacity is realized through an integrated high-stiffness ring base. By combining axisymmetric modes of ring base with the non-axisymmetric arrangement of feet, agile motions are achieved through a tripodal configuration while avoiding the over-positioning issues associated with quadrupedal designs. Furthermore, wireless motions are achieved through the integration of a power supply, allowing for rapidity and agile motions under high load capacity. Specifically, the proposed robot achieves the following advancements:

1. The tripodal robot features a simple, compact ring structure and a non-axisymmetric arrangement of 3 feet. High load capacity is achieved based on a high stiffness ring structure. By combining axisymmetric modes of ring base with the non-axisymmetric arrangement of 3 feet, multidimensional actuation trajectories at the foot ends are achieved, enabling the robot to achieve planar agile motions.

2. A compact robot was developed with a small size (30 × 30 × 7.6 mm^3^, and 38 × 38 × 15.5 mm^3^ after encapsulation) and weight (5.1 g for the robot and less than 10 g after encapsulation). The load capacity was achieved at 400 g (over 70 times the weight of the robot and over 40 times its weight after encapsulation).

3. By employing continuous and pulsed ultrasonic excitation schemes, high speed and high resolution were achieved in wired motions. The maximum speeds for linear and rotational motions were recorded at 338 mm/s (about 9 BL/s) and 944°/s (157 RPM), respectively. The resolutions for linear and rotational motions were achieved at 0.81 μm and 36.14 μrad, respectively.

4. A compact integrated power supply was developed, enabling wireless motions of the robot (38 × 39 × 45 mm^3^). The untethered robot exhibits a maximum linear and rotational speeds of 93 mm/s and 438°/s, and load capacity reaches 200 g, while the resolution is 0.63 μm. This feature eliminates the constraints of external wiring, further enhancing the robot’s mobility.

## Results

### Configuration design

**Fig. 1.**
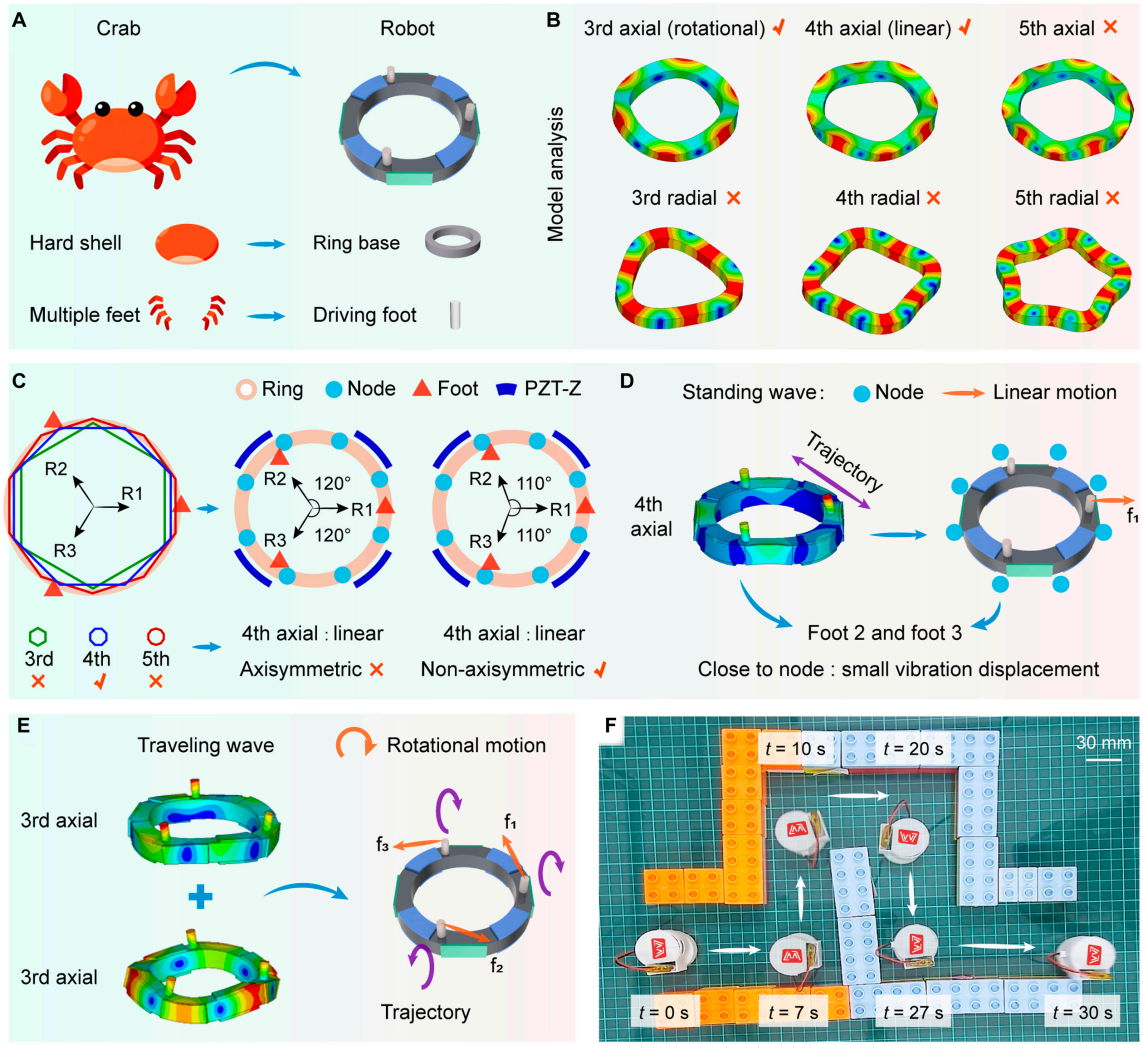
The configuration design, principles, and motion display of the robot. (A) The structures are inspired by crab. (B) The different modes of the ring structure; the modes mainly include the third- and fourth-order axial and radial modes. (C) Different modal node positions and different configurations of driving feet in a ring structure; make sure the driving foot is located far away from the node position, and the non-driving foot is positioned near the node. (D) The principle of linear motion. (E) The principle of rotational motions. (F) Display of robot motion trajectory; it can successfully traverse a trajectory through linear and rotational motions.

Inspired by the hard-shelled animals in nature, such as crabs, this work presents a ring-shaped tripodal piezoelectric robot with non-axisymmetrically arranged feet. The bionics can be elaborated on from the following 2 aspects. Firstly, the rigid ring structure proposed by imitating hard shells can enhance the load capacity of the robot. Crabs possess hard shells that can protect their bodies and assist them in bearing heavy loads. Inspired by this biological characteristic, an integrated rigid ring structure has been proposed, featuring high stiffness and large load capacity. Secondly, the multidimensional trajectories at the foot ends proposed by imitating multilegged creatures enable the robot to achieve agile motions. Crabs can achieve free and agile motions on the ground through the coordination of their multiple legs. Focusing on the coordination of multiple legs, different vibration modes of the ring structure have been selected to synthesize multidimensional trajectories at the ends of the multiple legs, thereby realizing linear and rotational motions, respectively. Based on the ring structure, the influence of different numbers of feet on the characteristics of robot has been analyzed (see Note S1 and Table S1). When the number of feet exceeds 3 (i.e., 4, 6, or even more), there is an over-positioning problem. Therefore, the relatively simple tripod configuration is selected. The proposed robot features a simple structure (see Fig. 1A), consisting of only 1 ring base, 3 feet, 4 radial piezoelectric ceramics (PZT-R, green color), and 8 axial piezoelectric ceramics (PZT-Z, blue color). For clarity, the driving feet are designated as foot 1 to foot 3, and a coordinate system O-RTZ is established at the end of each foot, with the corresponding radial direction defined as the R direction, the Z direction oriented vertically upward, and the T direction determined according to the right-hand rule. The polarization directions of ceramics are shown (for more details, see Fig. S1 and Movie S1).

It is necessary to choose the appropriate modes and determine the size and spacing of feet reasonably. To ensure that the working frequency of the robot is greater than 20 kHz, the first-order and second-order modes of the circular ring structure are not selected. Higher-order modes are generally not considered for their smaller amplitudes; thus, the third-order to fifth-order modes are displayed (see Fig. 1B). To excite traveling waves to achieve rotational motions, 2 standing waves with a spatial difference of 1/4 wavelength are required; therefore, 4 sets of PZT-Z circumferential arrangements are selected. The third-order and fifth-order axial modes can be used, as mentioned before, and the third-order axial modes are selected to obtain greater vibration amplitudes. When exciting the traveling wave, the positions of the nodes (the positions that remain stationary during vibration) are changing, and there is no need to consider the position relationship between the driving feet and the nodes. For linear motion, the arrangement of feet is determined by the positions of nodes. Taking the axial mode as an example, its displacement (*w*) can be simply expressed (see Eq. S1). The angular positions of the nodes (*θ*_node_) can also be expressed (see Eq. S2). The vertices of different polygons represent nodes of different order modes. The direction of linear motion is the R1 direction; thus, foot 1 should be located far away from the node position, and foot 2 and foot 3 should be positioned near the node. It can be noted that the fourth-order modes meet the requirements in several modes. The Z direction amplitude of the fourth-order radial mode is relatively small, making it difficult to overcome the roughness of the glass surface; thus, the fourth-order axial mode is used.

The size of the feet mainly affects the modal frequencies and displacements of foot end, thereby exerting an influence on vibration coupling and motion efficiency. Specifically, the rotational motions of the robot are achieved by exciting 2 third-order axial modes to synthesize traveling waves, which requires the frequency degeneracy of the 2 third-order axial modes (with an error of less than 3%). The finite element method (FEM) was conducted to determine it (see Fig. S2). The spacing of the feet mainly affects the modal frequencies, the relative positions between the driving feet and nodes, and the stability of robot, thereby exerting an influence on vibration coupling and motion efficiency. The selection of the angle is the result of a comprehensive consideration of the degenerate frequencies of the third-order axial modes (for achieving high-efficiency rotational motions), the relative positions between the driving feet and nodes (for achieving high-efficiency linear motion), and motion stability. Ultimately, a non-axisymmetric arrangement (110°) was considered (see Fig. S2 and Note S2).

Compared to the axisymmetric arrangement, foot 2 and foot 3 under the non-axisymmetric arrangement are closer to the nodes and do not interfere with PZT-Z. Rotational motions are achieved by exciting traveling waves of 2 third-order axial modes; at this point, the non-axisymmetric arrangement still meets the requirements. The trajectories and force situations of the foot ends under linear and rotational motions are displayed, respectively (see Fig. 1D and E). In our previous work, a quadruped ring-shaped piezoelectric robot with axisymmetric layout was proposed [40]. It can achieve agile motions. However, the quadruped robot requires additional weight to ensure 3 feet can touch the ground and achieve better motions, and it also does not achieve wireless motions. The proposed robot does not require additional weight. The robot can traverse a trajectory in wireless motions, highlighting its flexibility in motions (see Fig. 1F).

### Working principle

**Fig. 2.**
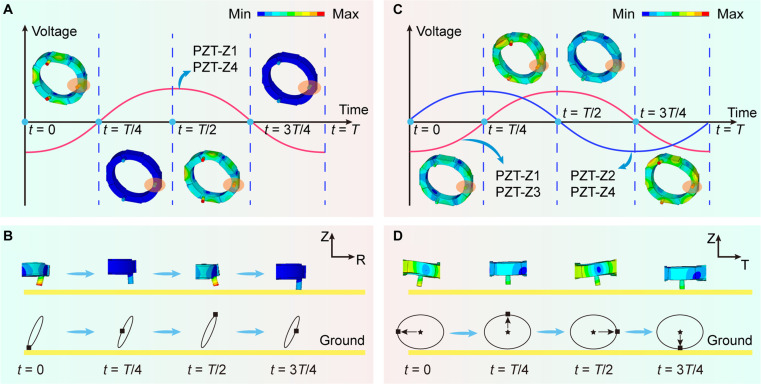
The working principles of the linear and rotational motions. (A) The linear motion of the robot within one cycle. (B) The motion of foot 1 within one cycle, and the displacement change of foot 1 in ROZ plane can be observed; the end of foot 1 is a diagonal trajectory. (C) The rotational motion within one cycle. (D) The motion of foot 1 within one cycle, and the displacement change in TOZ plane can be observed; the end of foot 1 is an elliptical trajectory.

During the linear motion, the trajectory of the foot end over one excitation cycle is illustrated (see Fig. 2A and B and Movie S2). To achieve a large displacement of foot 1, PZT-Z1 and PZT-Z4 near foot 1 are excited. By applying an excitation signal to PZT-Z1 and PZT-Z4, the fourth-order axial vibration mode can be excited, resulting in the generation of a diagonal trajectory at the foot end with actuating effects. Four specific positions of foot 1 are selected for illustration. Assuming that the duration of the excitation cycle is *T*, the 4 specific moments are *t* = 0, *t* = *T*/4, *t* = *T*/2, and *t* = 3*T*/4, respectively. The generation of the trajectory at the foot ends is illustrated by taking the vibration modes at the above 4 specific moments as examples. After one excitation cycle, the end of foot 1 can achieve a diagonal trajectory. When foot 1 moves to the bottom of the diagonal trajectory and contacts the ground, relative motion occurs with the ground, resulting in a frictional force acting in the opposite direction, thereby facilitating linear motion in the R1 direction. Through the accumulation of displacements over consecutive cycles, larger stroke motions can be achieved.

Similarly, during the rotational motions, the trajectory of the foot end over one excitation cycle is illustrated (see Fig. 2C and D and Movie S2). By applying 2 excitation signals with a phase difference of 90° to PZT-Z1 and PZT-Z3, as well as to PZT-Z2 and PZT-Z4, the third-order axial vibration modes of the robot can be excited. The circumferential traveling wave is synthesized by these excited third-order axial vibration modes, which subsequently generates elliptical trajectories with actuating effects at the foot ends. Rotational motion in one direction can be achieved through friction between the foot and the ground, and by altering the phase of the excitation signal to −90°, reverse rotational motion can be realized.

### Simulation analysis and parameter determination

**Fig. 3.**
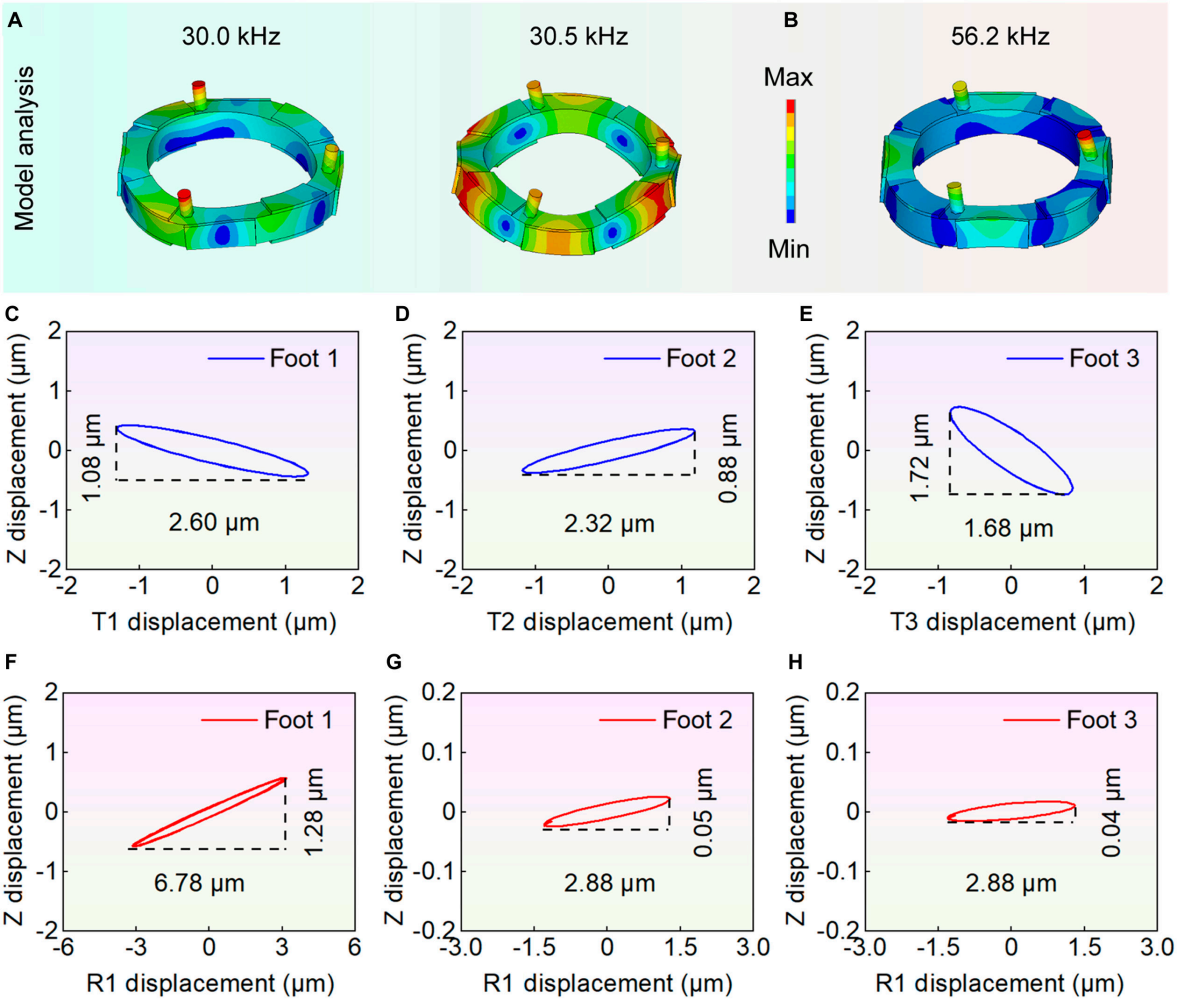
The analysis results of the FEM. (A) The third-order axial vibration modes, with frequencies of 30.0 and 30.5 kHz, respectively, and the difference is less than 0.5 kHz (error: 1.67%). (B) The fourth-order axial vibration mode, with a frequency of 56.2 kHz. (C) to (E) represent the trajectories of foot 1 to foot 3, in the rotational motions (third-order axial vibration modes), and the trajectories in the TOZ plane are all ellipses. (F) to (H) represent the trajectories of foot 1 to foot 3, in the linear motion (fourth-order axial vibration mode), and the displacement of foot 1 in R1 direction is 6.78 μm.

Determination of key parameters were shown (see Note S2). The modal analysis results of the robot were presented (see Fig. 3A and B and Movie S3). Among them, the 2 third-order axial modes (30.0 and 30.5 kHz) exhibited a frequency difference of approximately 0.5 kHz, corresponding to an error of 1.67%, which could be used for achieving rotational motions. The fourth-order axial mode (56.2 kHz) could be utilized to realize linear motion. The results of transient analysis were as follows: take the rotational motion in one direction as an example, 2 excitation signals with a 90° phase difference were applied to PZT-Z1 and Z3, as well as to PZT-Z2 and Z4, both at a frequency of 30.3 kHz and a voltage of 100 V_p-p_. The results (see Fig. 3C to E) indicated that the foot ends followed an approximately elliptical trajectory, with the displacements of foot 1, foot 2, and foot 3 in the T direction measured as 2.60, 2.32, and 1.68 μm, respectively, meeting the design requirements. The displacements of the 3 feet in the T direction were inconsistent, which may be caused by the non-axisymmetric structure; trajectory deviations were tested to evaluate the errors. For linear motion in the R1 direction, when the fourth-order axial mode was excited, a signal with a frequency of 56.2 kHz and a voltage of 100 V_p-p_ was applied to PZT-Z1 and PZT-Z4. The displacements of foot 1, foot 2, and foot 3 in the R1 direction were 6.78, 2.88, and 2.88 μm, respectively (see Fig. 3F to H). The Z direction displacements of foot 2 and foot 3 were relatively small, resulting in minimal effect to linear motion. Thus, linear motion in the R1 direction could be achieved by R1 displacement of foot 1.

### Experiments of the prototype

**Fig. 4.**
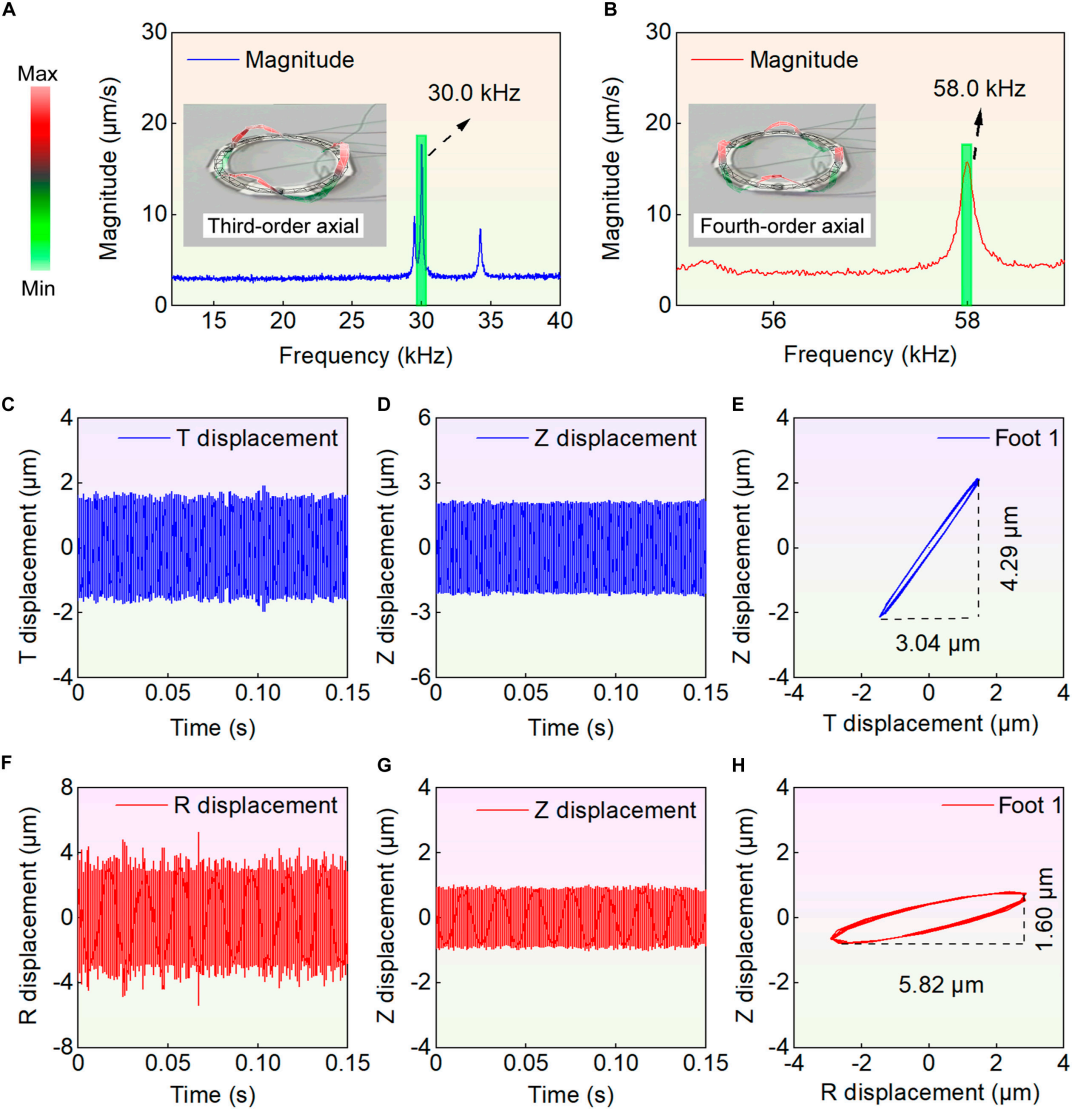
Vibration characteristics and foot trajectories of the robot. (A) and (B) are the vibration velocity response of the third and fourth axial vibration bending modes, with frequencies of 30.0 and 58.0 kHz, respectively, and there are vibration shapes of the third axial and the fourth axial vibration modes; red and green represent the outward and inward deformation of the tested position perpendicular to the measured surface, respectively. (C) to (E) are the displacement and trajectory of foot 1 in rotational motions, the displacement in the T1 direction is 3.04 μm, and the displacement in the Z1 direction is 4.29 μm. (F) to (H) are the displacement and trajectory of foot 1 in linear motion, the displacement in the R1 direction is 5.82 μm, and the displacement in the Z1 direction is 1.60 μm.

The vibration characteristics of the robot were presented (see Fig. 4A and B and Movie S4). Two axial modal shapes were measured experimentally, with the frequency of the third-order axial mode recorded at 30.0 kHz and the frequency of the fourth-order axial mode at 58.0 kHz. A comparison of these experimental results with the simulation data was presented (see Table S2). The frequencies obtained from the measurements exhibited certain discrepancies with the simulation results. The frequency difference may be caused by manufacturing errors, assembly errors, and differences in boundary conditions. These differences were quantified through simulation analysis (see Fig. S5A). Among these factors, the manufacturing errors were regarded as exerting a dominant influence. Assembly errors also contributed to frequency difference, the adhesive layer was specifically ignored, and deviations existed between the actual bonding position and the ideal position of the ceramics. The impact of boundary condition differences on the frequency was relatively minor. However, the errors were all within 5%. Overall, the vibration measurement experiment successfully captured the required 2 modal shapes, thereby validating the effectiveness of the simulation analysis.

The foot trajectory was subsequently tested. Taking the R1 direction as an example, an excitation frequency of 58.0 kHz and an excitation voltage of 100 V_p-p_ were set, resulting in displacement data for foot 1. After filtering, data from 10 cycles were selected for presentation. The displacements in the 2 directions were combined to obtain the foot-end trajectory in the ROZ plane (see Fig. 4C to E). The displacements of foot 1 in the R1 and Z1 directions were measured to be 5.82 and 1.60 μm, respectively. Similarly, the trajectory of foot 1 in the TOZ plane during rotational motion in one direction was obtained (see Fig. 4F to H). Under an excitation frequency of 30 kHz and an excitation voltage of 100 V_p-p_, the displacements of foot 1 in the T1 and Z1 directions were recorded as 3.04 and 4.29 μm, respectively. A comparison of these experimental results with the simulation displacements was shown (see Table S3). A displacement difference was observed between the simulation and the experiment. These differences were quantified through simulation analysis (see Fig. S5B). The assembly errors exerted a relatively dominant influence. Deviations existed between the actual position and the ideal position of the ceramics. The connection position between the shell and robot was designed to be located at the vibration node, while minor deviations existed in the actual connection position. It was also partially attributed to manufacturing errors of ring and foot. Moreover, the laser spot has a certain diameter, leading to a deviation between the actual measurement position of the laser spot and the position in the simulation. Boundary conditions also exerted a certain influence on the displacement. Despite the mentioned differences, the measured displacement still meets the requirements. The results indicated that the desired trajectory was achieved at the driven foot end, thereby validating the feasibility of the principle, and the displacements also met the required specifications.

**Fig. 5.**
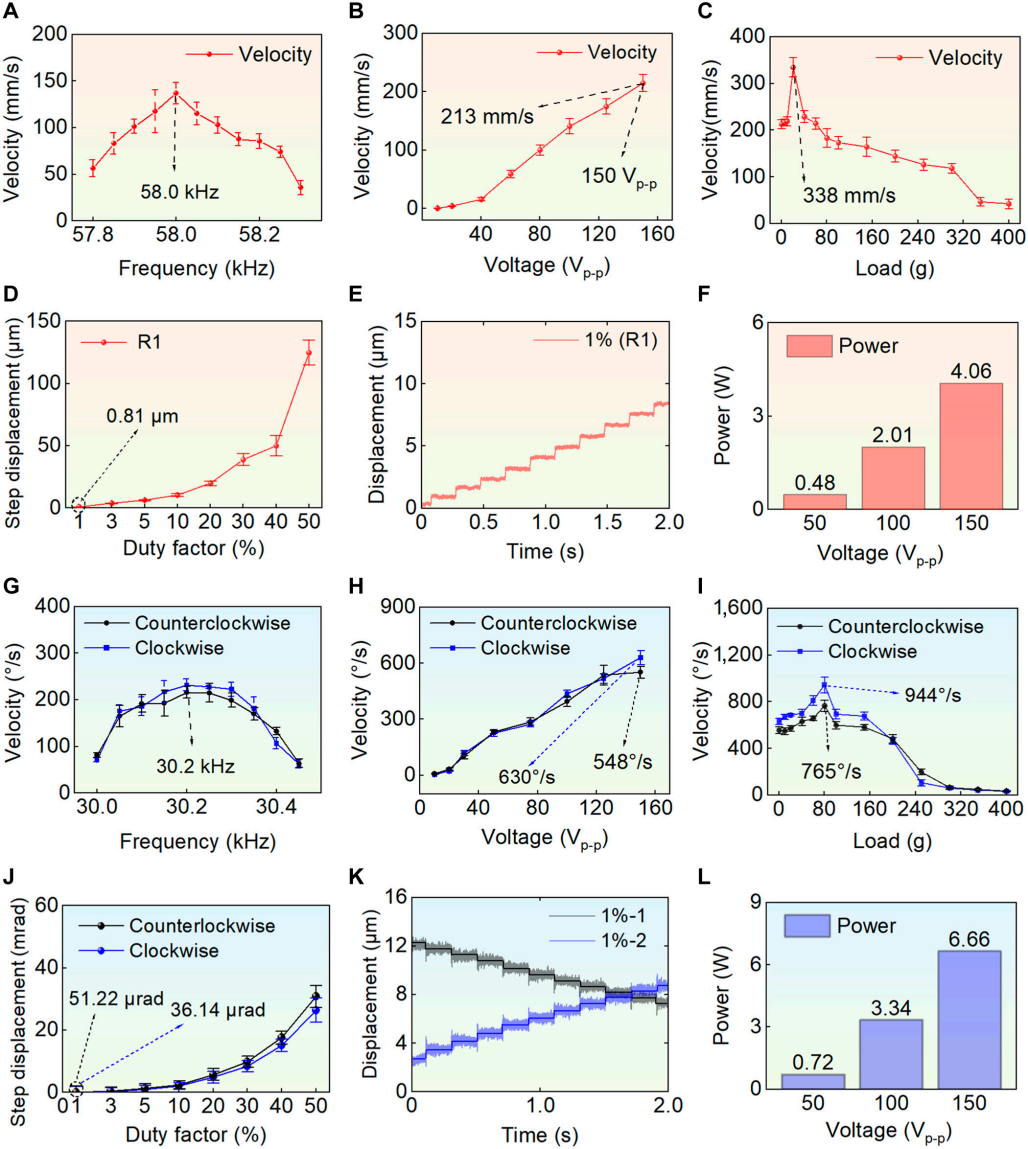
Wired motion characteristics of the robot; (A) to (F) are the characteristics of linear motion (red background), and (G) to (L) are the characteristics of rotational motions (blue background). (A) Relationship between the velocity and the frequency, and the optimal excitation frequency is 58.0 kHz. In the experiments, each data was tested 5 times (*n* = 5), and the mean and standard deviation were calculated to plot the error bars. (B) Relationship between the velocity and the voltage; when the voltage is 150 V_p-p_, the maximum linear motion speed is 213 mm/s. (C) Relationship between the velocity and the load (load capacity); when the load is 20 g, the speed reaches its maximum of 338 mm/s. (D) Step displacement of the R1 direction with duty factor from 1% to 50%; the resolution is 0.81 μm. (E) Displacement of the R1 direction with the duty factor of 1% (resolution). (F) Power consumptions in linear motion (under 50, 100, and 150 V_p-p_), at the maximum voltage (150 V_p-p_); the total power consumption is 4.06 W. (G) Relationship between the rotational speed and the frequency, and the optimal excitation frequency is 30.2 kHz. (H) Relationship between the rotational speed and the voltage; when the voltage is 150 V_p-p_, the maximum speed is 630°/s (clockwise) and 548°/s (counterclockwise). (I) Relationship between the rotational speed and the load (load capacity); when the load is 80 g, the speed reaches its maximum of 765°/s (counterclockwise) and 944°/s (clockwise). (J) Step displacement of rotational motions with duty factor from 1% to 50%; the resolution is 51.22 μrad (counterclockwise) and 36.14 μrad (clockwise). (K) Displacement of rotational motion with the duty factor of 1%. (L) Power consumptions (50, 100, and 150 V_p-p_); at the maximum voltage (150 V_p-p_), the total power consumption is 6.66 W.

The linear motion characteristics were tested (see Movie S5). The relationship between frequency and velocity was illustrated (see Fig. 5A). During the experiment, an excitation voltage of 100 V_p-p_ was applied, and the optimal excitation frequency for the prototype was determined to be 58.0 kHz, at which point the velocity was measured to be 136 mm/s. This frequency was subsequently used as the optimal excitation frequency for linear motion. At the optimal excitation frequency of 58.0 kHz, the excitation voltage was varied to obtain the velocity (see Fig. 5B). It could be observed that the velocity increased with the rise in voltage, reaching a maximum linear motion speed of 213 mm/s at 150 V_p-p_.

The load characteristics were tested. The maximum excitation voltage was set to 150 V_p-p_, while maintaining the optimal excitation frequency of 58.0 kHz, to measure the speed under different load conditions (see Fig. 5C). As the load increased, the speed exhibited an overall trend of first increasing and then decreasing. The reason is that an appropriate load increases the friction between the robot feet and the ground, thereby facilitating better motions of robot, whereas excessively high friction, in turn, hinders the motions of robot. In addition, drag force experiments under different load conditions were conducted. The magnitude of friction was intuitively demonstrated by the tension meter, thus verifying that the friction increased with the load increased (see Fig. S11A). The maximum speed of 338 mm/s was achieved at a load of 20 g. The linear speed under a load of 400 g (over 70 times the weight of robot and over 40 times its weight after encapsulation) was 28 mm/s. The experimental results indicated that the proposed robot possessed high stiffness and strong load capacity.

The resolution experiments were conducted. Resolution refers to the minimum stable motion step size measured experimentally, while the step size is determined by the number of vibration cycles of the foot end. The number of vibration cycles is governed by the frequency of the AC excitation signal, duty cycle, and pulse frequency (see Eq. S3). Pulse frequency refers to the frequency of the applied pulse signals. The duty cycle is defined as the ratio of the actual signal application time to the total period within each pulse signal cycle (see Fig. S8A). The frequency of the excitation signal was fixed at the optimal excitation frequency of 58.0 kHz, and the minimum duty cycle of the power supply was set to 1%. We gradually adjusted the pulse frequency until the minimum stable step size was measured. Ultimately, a pulse frequency of 5 Hz was selected (see Fig. S8). Varying the duty cycle from 1% to 50%, the step displacement was tested with a sampling frequency set at 5 kHz (see Fig. S9) and a pulse frequency of 5 Hz. For linear motion, the step displacement was directly measured along the direction of motion using a laser displacement sensor. Ten consecutive step distances were selected to calculate the step size in the R1 direction for each duty cycle. As the duty cycle increased, the step size also increased (see Fig. 5D). The displacement in the R1 direction at a duty cycle of 1% was illustrated, where the minimal step size corresponds to the resolution, calculated to be 0.81 μm (see Fig. 5E). This indicates that the robot achieves sub-micrometer resolution, demonstrating a high level of resolution. The power consumptions were tested using a power meter during linear motion excitation. It was measured at different voltages (see Fig. 5F). When the voltages were set to 50, 100, and 150 V_p-p_, the total power consumptions were around 0.48, 2.01, and 4.06 W, respectively. These results indicate that robot has a low power consumption, making it efficient for its operational tasks.

The rotational motion characteristics were similarly investigated (see Movie S5). Initially, the relationship between frequency and velocity was tested to determine the optimal excitation frequency. The excitation voltage was uniformly set to 50 V_p-p_, and all PZT-Z actuators were activated. By varying the excitation frequency, the velocity was obtained (see Fig. 5G). Each measurement was repeated multiple times and averaged to ensure accuracy. Based on data analysis, the optimal excitation frequency was found to be approximately 30.2 kHz. At this frequency, the clockwise and counterclockwise rotational speeds were measured to be 258°/s and 225°/s, respectively. The data curve indicates that there is a certain discrepancy (12.8%) between the clockwise and counterclockwise rotational speeds. The reason may be the non-axisymmetric structure, which results in the inability to excite perfect traveling waves in the ring base. Furthermore, small deviation of the rotation center was observed during the testing process.

Similarly, the voltage was gradually increased to a maximum of 150 V_p-p_, and the velocity was calculated (see Fig. 5H). Each measurement was repeated multiple times and averaged to ensure accuracy. It can be observed that the speed increases gradually with the increase in voltage. At the optimal excitation frequency of 30.2 kHz, the rotational speeds under 150 V_p-p_ voltage excitation reached 630°/s and 548°/s for clockwise and counterclockwise rotations, respectively. The speeds under different loads were illustrated (see Fig. 5I). As the load increased, the speed initially increased and then decreased. At a load of 80 g, the maximum rotational speeds were achieved, reaching 944°/s and 765°/s for clockwise and counterclockwise rotations, respectively. A certain discrepancy of 20.1% was noted between the clockwise and counterclockwise speeds, which may be attributed to the non-axisymmetric configuration, preventing the generation of a perfect traveling wave. An appropriate load can enhance the motion speed. The clockwise and counterclockwise speeds under a load of 400 g (over 70 times the weight of the robot and over 40 times its weight after encapsulation) were 5°/s and 6°/s, respectively, demonstrating that the robot possessed a strong load capacity.

Trajectory deviations under heavy load conditions (200, 300, and 400 g) were conducted. There were deviations during linear and rotational motions. The maximum deviation (coupled displacement in the vertical motion direction) of rotational motion was 28 mm, and for linear motion, the deviation was 21 mm when the robot moved 250 mm; the relative error was 8.4% (see Fig. S7).

The step displacement experiments were conducted, and the sampling frequency was set to 5 kHz, with a pulse frequency of 5 Hz and a duty cycle ranging from 1% to 50%. Two laser displacement sensors were employed to test the resolution. The resolutions during rotational motions cannot be obtained directly from the data. For rotational motions in pulse mode, when the rotation angle is small, the center of the robot may experience a certain offset during motion. Assuming the center shifts from *O*_1_ to *O*_2_, the schematic can be seen in Fig. S5. The rotational resolutions of the robot can be approximately obtained by Eqs. S4 and S5 (for more details, see Fig. S6C and Notes S3 and S4). The resolutions of the rotational motions were obtained (see Fig. 5J and K). In these figures, the motions in both clockwise and counterclockwise directions at a 1% duty cycle were displayed. Ten specific steps were selected for analysis, resulting in a resolution of 36.14 μrad for clockwise rotation and 51.22 μrad for counterclockwise rotation, respectively. The step displacement increased with the duty cycle increased.

Climbing experiments at different inclination angles were conducted. In the experiment, one side of a glass plate was elevated to form a slope, and the slope angle was adjusted by modifying the height of the padding. As indicated by the experimental results, the robot could climb a slope with an angle of approximately 5°. Meanwhile, moving experiments of robot on surfaces of different materials were conducted. The robot could move on the surfaces of marble, steel plate, and acrylic, demonstrating that the robot had a certain degree of adaptability (see Fig. S12).

During the excitation of the rotational motion in one direction, the power consumptions were tested. The rotational motion requires 2 signals, and the total power was defined as the sum of the powers from both circuits. Power measurements were conducted at voltage levels of 50, 100, and 150 V_p-p_ (see Fig. 5L). It was found that the power consumptions were approximately 0.72 W at 50 V_p-p_, around 3.34 W at 100 V_p-p_, and around 6.66 W at 150 V_p-p_. These results indicate that the robot exhibits low power consumption during rotational motion.

**Fig. 6.**
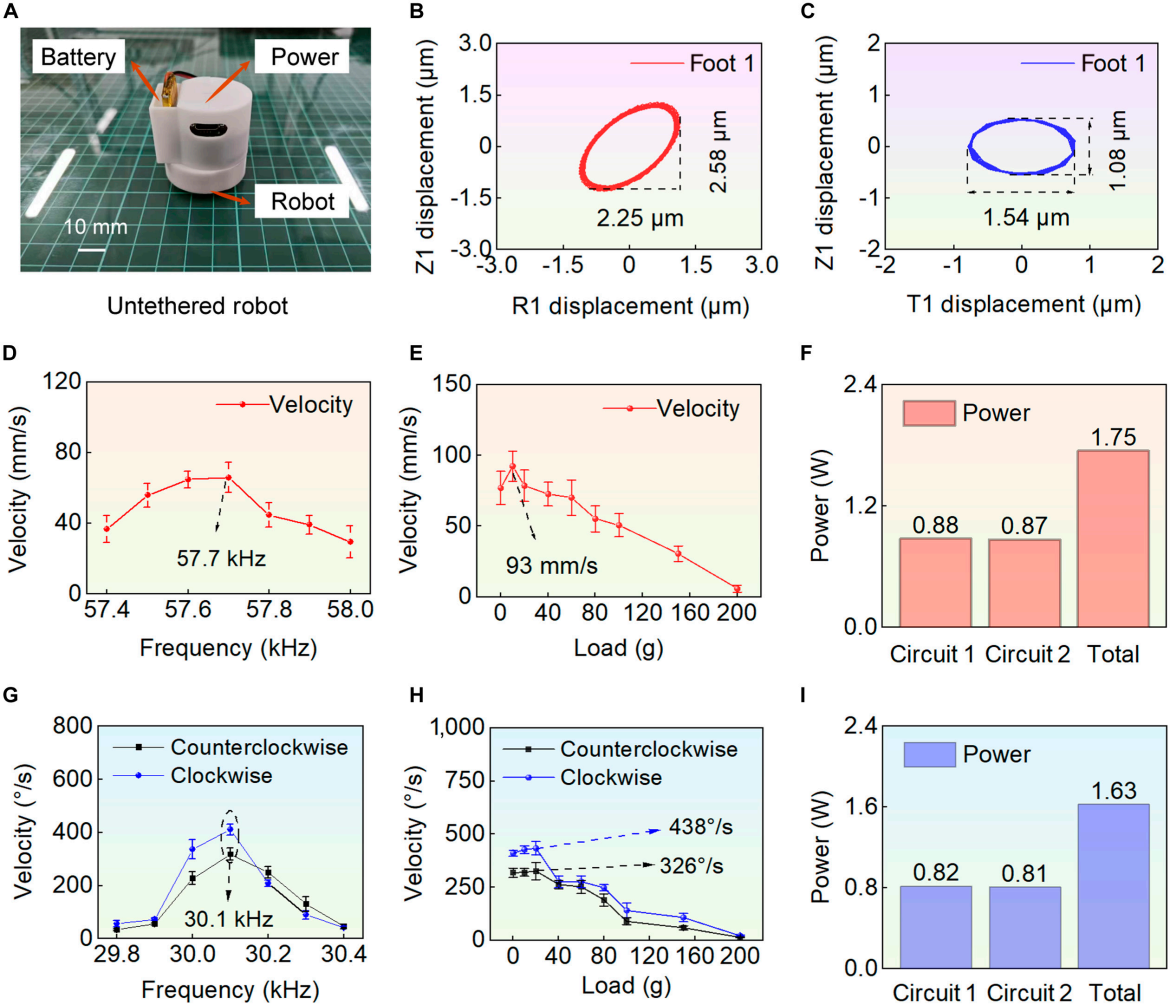
Wireless motion characteristics of robot. (A) Prototype of wireless robot. (B) Trajectory of foot 1 in linear motion, the displacement in the R1 direction is 2.25 μm, and the displacement in the Z1 direction is 2.58 μm. (C) Trajectory of foot 1 in rotational motion, the displacement in the T1 direction is 1.54 μm, and the displacement in the Z1 direction is 1.08 μm. (D) to (F) are the characteristics of linear motion (red background), and (G) to (I) are the characteristics of rotational motions (blue background). (D) Relationship between the velocity and the frequency, and the optimal excitation frequency is 57.7 kHz. (E) Relationship between the velocity and the load (load capacity); when the load is 10 g, the speed reaches its maximum of 93 mm/s. (F) The total power consumption is 1.75 W. (G) Relationship between the rotational speed and the frequency, and the optimal excitation frequency is 30.1 kHz. (H) Relationship between the rotational speed and the load (load capacity); when the load is 20 g, the speed reaches its maximum of 326°/s (counterclockwise) and 438°/s (clockwise). (I) The total power consumption is 1.63 W.

To further enhance the mobility and eliminate the constraints of external wiring, a compact integrated power supply was developed and designed in conjunction with the robot (see Fig. 6A). The control chip of wireless power supply is the ESP32, which is equipped with powerful PWM (pulse-width modulation) functionality. PWM waveforms with a voltage range of 0 to 3.3 V can be output by the GPIO (general purpose input/output) pins of the ESP32 chip, and after comparison by an operational amplifier, driving signals can be generated. In the experiment, commands are sent from the host computer (mobile phone) to the ESP32, enabling different GPIO pins to be controlled to output the corresponding PWM waveforms in real time, thereby realizing the linear and rotational motions of robot. More details are shown (see Fig. S10 and Note S5 [41]). The overall dimensions of the integrated system are approximately 38 × 39 × 45 mm^3^, and when powered by a 200-mA·h lithium battery, the total weight is about 28.5 g. Subsequently, experiments were conducted to evaluate certain wireless characteristics of robot with the integrated power supply.

The displacement and trajectory of the foot end were measured to verify the motion principle. Taking foot 1 as an example, when the excitation frequency was set to 58.0 kHz and the excitation voltage to 104 V_p-p_ (linear motion), the displacements of the 3 feet in the OR and OZ directions were measured. After filtering, the trajectories in the ROZ plane were plotted (see Fig. 6B). The displacements of foot 1 in the R1 and Z1 directions were 2.25 and 2.58 μm, respectively. For foot 2, the displacements in the R1 and Z1 directions were 0.94 and 0.65 μm, respectively. The displacements in the R1 and Z1 directions of foot 3 were 1.22 and 0.66 μm, respectively. In the R1 direction, a displacement difference was observed between foot 1 and foot 2 and 3; the difference was relatively small. Therefore, the linear motion speed was found to be reduced compared to wired operation.

When the excitation frequency was set to 30 kHz and the excitation voltage was set to 104 V_p-p_ (rotational motion), the end displacement data were obtained. After filtering, the trajectory in the TOZ plane was plotted (see Fig. 6C). The displacements of foot 1 in the T1 and Z1 directions were 1.54 and 1.08 μm, respectively. The results indicate that in both cases, the desired elliptical trajectories at the end of the driving feet were successfully achieved. However, the motion speed was relatively reduced compared to wired operation. Nevertheless, the feasibility and demonstrability of wireless operation are the primary concerns, and the speed is not a major requirement.

It could be observed that under wireless mode, the optimal excitation frequency of linear motion was 57.7 kHz (see Fig. 6D to F). The frequency of wireless motion is slightly different from wired motion due to the integration of a driving power supply, resulting in changes in the overall structure and quality of the prototype. At the optimal excitation frequency, the maximum speed of wireless motion was 93 mm/s with a load of 10 g. For rotational motions (see Fig. 6G to I), the optimal excitation frequency was 30.1 kHz. At a frequency of 30.1 kHz, the maximum speeds were 438°/s and 326°/s for clockwise and counterclockwise rotations, respectively, at a load of 20 g (see Movie S6). The trend of the speed curve is the same as before. The power consumption was 1.63 W. Under the same experimental conditions, the wired speeds remained higher than the wireless ones, with the output power of the wireless power supply serving as the primary limiting factor under this circumstance. When the excitation voltage was set to 100 V_p-p_, the power consumption of wired motion was 2.01 W, whereas that of wireless motion was 1.75 W. The limited output power of the wireless power supply restricted the motion speeds of robot. In future work, the wireless power supply scheme can be optimized to achieve higher power output.

Trajectory deviations of untethered robot under a load of 200 g were conducted. The maximum deviations (coupled displacement in the vertical motion direction) of rotational and linear motions were both 19 mm (when the robot moved 200 mm, and the relative error was 9.5%, see Fig. S7 and Movie S7). Climbing experiments under wireless motion were also conducted. As indicated by the experimental results, the untethered robot could climb a slope with an angle of approximately 2° (see Fig. S12 and Movie S8). The untethered robot exhibited inferior climbing capability, which was restricted by the output voltage and power of the wireless power supply. In addition, the integration of the wireless power supply raised the center of mass of the robot, thereby reducing its climbing performance. Meanwhile, moving experiments of robot on surfaces of different materials were conducted. The untethered robot could move on the surfaces of marble, steel plate, and acrylic, demonstrating that the robot had a certain degree of adaptability (see Fig. S12 and Movie S9).

The endurance time of the robot was tested while it continuously performed rotational motions. When powered by a 200-mA·h lithium battery, the robot exhibited an endurance time of approximately 20 min. When powered by a 500-mA·h lithium battery, the endurance time was about 55 min, and the total weight is 33.1 g. The lithium battery can be selected according to specific requirements. Noise experiments were conducted, in which the acoustic noise level of robot during operation was measured. Three special moments (the starting noise, maximum noise, and ending noise during operation) were selected, and the maximum noise variation of the robot during operation was not above 1.6 dB (see Fig. S12A and B and Movie S10). The noise of the robot was low due to the ultrasonic working frequency.

**Fig. 7.**
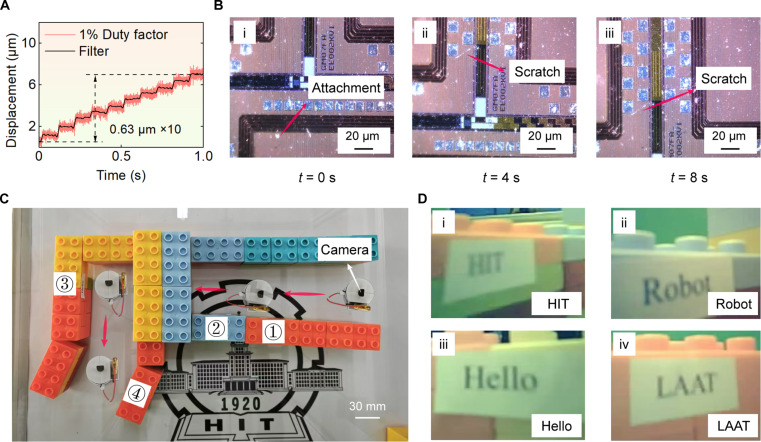
The application experiments of the robot: the wafer detection experiment and object detection experiment. (A) The displacement under the excitation signal with a 1% duty cycle; a total of 10 steps were selected, resulting in a total displacement of 6.3 μm, which yields an average step size of 0.63 μm, representing the resolution in wireless mode. (B) (i) to (iii) show the detection of a wafer, the wafer was fixed on the robot, and a microscope (1,000×) was used to detect the wafer. (C) The motion trajectory of the robot with a camera; it can recognize the texts affixed to the sidewalls of the blocks. (D) (i) to (iv) show the recognition of the texts “HIT”, “Robot”, “Hello”, and “LAAT”.

Under the excitation of pulse signals, the robot was enabled to perform stepping motion with micrometer-level step sizes. By varying the duty cycle, the robot was able to move with different step sizes. Specifically, under the excitation signal with a 1% duty cycle, the displacement was obtained (see Fig. 7A). A total of 10 consecutive steps were selected, resulting in a total displacement of 6.3 μm, which yielded an average step size of 0.63 μm. Thus, the resolution of the wireless motion was measured to be 0.63 μm, with the 1 σ repeatability of 0.09 μm. The resolution of wireless motion was better than that in wired motion, the reason may be that the wireless power supply acted as a load, and the friction at foot ends could be increased by the load, thereby changing the resolution of the robot. The load-resolution experiment was performed to demonstrate it; more details could be seen in Note S6. As a result, an appropriate load could enhance resolution; meanwhile, the robot could still maintain micrometer-level resolution even under heavy load conditions (see Fig. S11B and C).

The wireless application experiments were conducted on the untethered robot to expand its application scenarios. The robot was set to move with micro steps and a wafer was fixed on it. At this point, a 1,000× microscope was utilized to inspect the wafer surface, allowing for the observation of scratches and contaminants on the surface, as depicted [see Fig. 7B(i) to (iii) and Movie S11]. Moreover, multiple wafer inspection experiments were conducted (a scale bar was labeled on the wafer images to facilitate the understanding of wafer dimensions). The start and end positions were designated, and deviations (coupled displacement in the vertical motion direction) of robot during multiple motions were plotted. The average deviation of the robot in the multiple motion experiments was 5.1 μm when the robot moved about 260 μm, and the relative error was 2.0% (see Fig. S13). This indicates that the robot possesses the capability for precise operations and inspections at the microscopic scale, thereby further expanding its potential applications in micro-nano operations and surface inspections.

A trajectory demonstration experiment was conducted. To facilitate testing, a trajectory was constructed using building blocks. As mentioned before, the robot successfully traversed the trajectory through linear and rotational motions (see Fig. 1F and Movie S12). Furthermore, the ESP32 chip, which is used for the integrated power supply, allows for the integration of a camera, resulting in a more compact design. Simple trajectories were built using the blocks, with the texts “HIT”, “Robot”, “Hello”, and “LAAT”, affixed to the sidewalls of the blocks. The robot successfully recognized the texts through the camera [see Fig. 7C and D(i) to (iv) and Movie S13].

The tethered and untethered characteristics are presented (see Table S4). In terms of maximum motion speed, the wireless motion speed is lower than that of the wired configuration. However, the power consumption is reduced, suggesting that this decrease in speed may be attributed to power limitations, indicating potential areas for targeted improvements in the future. The untethered robot can achieve a resolution of 0.63 μm, which is better than that of the tethered one. The development of the wireless power supply is primarily aimed at enhancing the mobility and flexibility, thereby freeing it from the constraints of wired connections.

A simple comparison between the tripodal robot in this work and the quadrupedal robot in the previous work [40] is presented. By selecting different vibration modes of ring base and various arrangements of driving feet, planar agile motions can be achieved by 2 robots. The quadrupedal robot can easily excite perfect traveling waves by the axisymmetric arrangement of feet, resulting in better rotation characteristics. The tripodal robot achieves higher linear velocity by the non-axisymmetric arrangement of feet. The driving foot is positioned far from the node while the other 2 feet are located close to the node. The 3 feet can contact the ground without additional weight. The tripodal robot in this work also integrates wireless power supply, achieving more agile wireless motions.

**Fig. 8.**
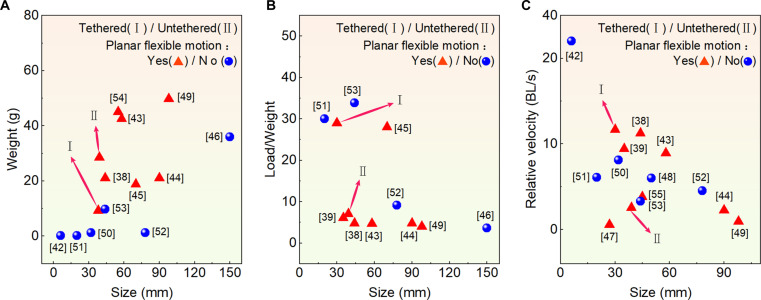
Comparison with other piezoelectric robots; red triangles and blue circles are used to represent whether planar agile motions can be achieved, I (tethered) and II (untethered) represent our work, and our robot balances small size, high load capacity, and planar agile motions. (A) The comparison of weight and size. (B) The comparison of load capacity and size. (C) The comparison of relative velocity and size.

A comparison with other piezoelectric robots of similar size is shown (see Fig. 8 and Table S5) [38,39,42–55]. The Milli-Walker [42] features a small size (6 × 2 × 2 mm^3^); its maximum speed is over 30 BL/s, but it cannot achieve rotational motion. The untethered PISCES [44] can achieve planar agile motions; however, it has a relatively large size (90 × 60 × 11 mm^3^), and the load capacity is relatively weak (100 g). The untethered TMPR developed by Li et al. [53] has a ratio of 33.8, while it cannot achieve planar agile motions (its rotational motion requires a guiding mechanism). The robot performs excellently in specific fields, such as achieving a load-to-mass ratio of 40, which is attributed to its rigid ring structure. The proposed robot balances small size, high load capacity, and planar agile motions, and can achieve wireless motions.

## Discussion

To address the challenge of balancing planar agile motions with high load capacity in high-speed miniature robots, we proposed a unique tripodal piezoelectric robot featuring a high-stiffness axisymmetric base and 3 non-axisymmetrically arranged feet. Through ultrasonic resonance, noiseless high-speed motions have been achieved; on this basis, the load capacity has been enhanced by the high-stiffness ring base. Through comprehensive analysis of vibration modes, as well as the number and layout of driving feet, multidimensional actuation trajectories of the foot ends have been obtained, enabling agile motions of robot. These designs were validated through FEM. Subsequently, a tethered prototype was produced. The results indicated that maximum speeds of 338 mm/s and 944°/s were reached in linear and rotational motions, with resolutions of 0.81 μm and 36.14 μrad, respectively. To further enhance the mobility and eliminate the constraints of wired connections, a compact integrated power supply was developed, enabling wireless operation. The untethered robot exhibited a maximum linear speed of 93 mm/s and a maximum rotational speed of 438°/s. The load capacity reached 200 g, and the resolution in wireless mode was 0.63 μm. The core innovation of this work lies in achieving both large load capacity and high flexibility by selecting the axisymmetric mode of the ring structure and arranging the driving feet asymmetrically. Meanwhile, a prototype has been developed for verification, demonstrating good performance. However, the current research is focused on open-loop experimental tests, and trajectory errors have not yet been corrected through feedback. In future work, visual feedback can be introduced to correct trajectory errors and further improve the motion performance of the robot. Moreover, the integration of gripping units and other components will be pursued to perform micro-manipulation tasks, further expanding the application scenarios.

## Materials and Methods

### Simulation analysis of robot

FEM (Model: APDL 17.1, ANSYS, USA) was carried out to determine structural parameters based on 3 key indicators. One is that the frequency of the third-order axial vibration modes should be as close as possible, with a difference of no more than 3%. They all need to be greater than 20 kHz to ensure that there is no noise during motion. The last one is that there must be an effective displacement at the foot end. Specifically, the displacement of driving foot should be greater than 1 μm, which can ensure that the robot has better motion performance. Based on 3 key indicators mentioned above, the parameters were determined through simulation analysis (see Figs. S1 and S2).

### Materials and fabrication

The robot consists of only 1 ring base, 3 driving feet, 4 radial piezoelectric ceramics, and 8 axial piezoelectric ceramics. The ring base is made of aluminum alloy 2A12 material, processed by a lathe. The driving foot is made of alumina ceramic material, which is wear-resistant, and the ceramic is made of PZT-4 material. The prototype assembly mainly has the following steps: cleaning the ring base and ceramics with ethanol, which can remove impurities from the surface. Both the driving foot and the ceramic were pasted on the ring base using epoxy resin. When the epoxy resin was completely solidified, the wire was welded to the ceramics to apply excitation signals (see Fig. S3).

### Measurement and characterization of robot

The vibration characteristics were tested using a scanning laser Doppler vibrometer (Model: PSV-400-M2, Polytec, Germany), which can check the vibration modes and corresponding frequencies (see Fig. S4). To verify the working principle of linear and rotational motions, a laser displacement sensor (Model: LK-H020, Keyence, Japan) was used to measure the motion trajectory of the end of driving foot. While a power supply (Model: made by our laboratory) was used to generate sinusoidal excitation signals. When testing the resolution characteristics, a laser displacement sensor was used to measure the motion steps, the steps can be obtained by processing the data (Model: Origin, OriginLab, USA), and the resolutions can be obtained (more details are shown in Fig. S6). When testing the other characteristics, a camera (Model: E14a, Hikvision, China) was used to shoot videos, then uses the Adobe Premiere software to process key frames to complete speed calculations. In the experiments, each data was tested 3 times, and the mean and standard deviation were calculated to plot the error bars. Power consumption was tested by using a digital power meter (WT210, Yokogawa, Japan). Scratches and attachments on the wafer were detected under a 1,000× magnification (Model: B011, Supereyes, China). Moreover, the ESP32 (Model: Seeed Studio XIAO ESP32S3, Seeed Technology, China) was used as the controller of the integrated power supply (see Fig. S10). The figures were plotted by Origin and Adobe Illustrator software and the movies were produced by Adobe Premiere Pro software.

## Data Availability

All data supporting the findings of this study are available in the paper and the Supplementary Materials.

## Supplementary Materials

Supplementary 1 Notes S1 to S6 Figs. S1 to S13 Tables S1 to S5 Movies S1 to S13
