# Supplementary material for: An Untethered Ring-Shaped Miniature Robot with Axisymmetric Vibrations and Non-axisymmetrically Arranged Feet
Source: Research (Wash D C). 2026 Feb 19;9:1158. doi: 10.34133/research.1158 (PMC12917124; doi:10.34133/research.1158)
Supplement: Supplementary 1 — Notes S1 to S6 Figs. S1 to S13 Tables S1 to S5 Movies S1 to S13 [file research.1158.f1.zip › Supplementary Information with highlight.docx]

Supporting Information

**An Untethered Ring-shaped Miniature Robot with** **Axisymmetric Vibrations and Non-axisymmetrically Arranged Feet**

Baoyi Liu†, Jing Li†, Yu Gao, Jinghan Guan, Boliang Xu, Jie Deng, Shijing Zhang, Yingxiang Liu*

†These authors contributed equally to this work.

*Corresponding author. Email: [liuyingxiang868@hit.edu.cn](mailto:liuyingxiang868@hit.edu.cn) (Y.L.).

**The Supporting Information includes:**

**Note S1.** Configuration design of robot.

**Note S2.** Determination of key parameters.

**Note S3.** The discussion about sampling frequency and pulse frequency.

**Note S4.** Calculation method for resolution of rotational motions.

**Note S5.** The scheme and characteristics of wireless power supply.

**Note S6.** The relationship between load and resolution.

**Fig. S1.** The structure of robot and polarization direction of ceramics.

**Fig. S2.** Relationship between various parameters and frequency difference.

**Fig. S3.** The structural parameters and prototype of robot.

**Fig. S4.** Test method for vibration characteristics of robot.

**Fig. S5.** The influence of different errors on frequency and displacement.

**Fig. S6.** Test methods for the motion characteristics of robot.

**Fig. S7.** Trajectory deviations of robot.

**Fig. S8.** The explanation of pulse signal and step displacement.

**Fig. S9.** The data under different sampling frequencies.

**Fig. S10.** The scheme, prototype and output characteristics of wireless power supply.

**Fig. S11.** The relationship between load and force, load and resolution.

**Fig. S12.** The noise, climbing ability and adaptability of robot.

**Fig. S13.** Wafer inspection experiment.

**Table S1.** Influence of different numbers of feet on the characteristics of robot.

**Table S2.** Comparison of simulation frequency with experimental frequency.

**Table S3.** Comparison of simulation displacement with experimental displacement.

**Table S4.** Comparison of tethered characteristics with untethered characteristics.

**Table S5.** Comparison of characteristics with other small robots.

**Other** **Supplementary Materials for this manuscript includes the following：**

**Movie S1.** Configuration of robot.

**Movie S2.** Working principles of linear and rotational motions of robot.

**Movie S3.** Simulation analysis of robot.

**Movie S4.** Vibration characteristics of robot.

**Movie S5.** Tethered motion characteristics of robot.

**Movie S6.** Untethered motion characteristics of robot.

**Movie S7.** Trajectory deviations of robot under heavy load conditions.

**Movie S8.** Climbing slopes of different angles.

**Movie S9.** Moving on different surfaces.

**Movie S10.** Acoustic noise level of the robot during operation.

**Movie S11.** Wafer detection experiments.

**Movie S12.** Untethered agile motions of robot.

**Movie S13.** Object identification experiments.

**Supplementary Notes**

**Note S1.** Configuration design of robot.

The bionics in this work tends more towards functional bionics, which is elaborated on from the following two aspects. Firstly, the rigid ring structure proposed by imitating hard shells can enhance the load capacity of robot. Hard-shelled animals in nature, such as crabs, possess hard shells that can protect their bodies and assist them in bearing heavy loads. Inspired by this, an integrated rigid ring structure has been proposed, which is characterized by high stiffness and the ability to bear large loads. Secondly, the multi-dimensional trajectories at the foot ends proposed by imitating multi-legged creatures enable the robot to achieve agile motions. Hard-shelled animals can achieve free and agile motions on the ground through the coordination of their multiple legs. Focusing on the coordination of multiple legs, inspired by this, different vibration modes of the ring structure have been selected to synthesize multi-dimensional trajectories at the ends of the multiple legs, thereby realizing linear and rotational motions respectively. Based on the ring structure, the influence of different numbers of feet on the characteristics of robot has been analyzed (see Table. S1). First, it is demonstrated that the stability is enhanced with the increase in the number of feet, and the load-bearing capacity is slightly improved accordingly. However, when the number of feet exceeds three (i.e., 4, 6, or even more), there are over-positioning problem, which generally requires additional loads to ensure full contact between multiple feet and the ground. In contrast, the tripod configuration is free from the over-positioning problem and thus no extra load needs to be applied. Meanwhile, the linear motion speed can be easily improved by arranging the positions of the three feet, with the driving feet placed away from the nodes and the non-driving feet close to the nodes. With the increase in the number of feet, it is difficult to arrange all non-driving feet close to the nodes. If the non-driving feet are placed away from the nodes, the motion of the driving feet will be affected, leading to an increase in the motion coupling between the feet, which in turn reduces the motion speed and efficiency of robot. In addition, the control system becomes more complex as the number of feet increases. Therefore, the relatively simple tripod configuration is selected, which is free from over-positioning problem. By combining different vibration modes of the ring and arranging three feet non-axisymmetrically, multidimensional actuation trajectories are generated at the foot ends, thereby enabling agile planar motions.

It is necessary to choose the appropriate modes and determine the size and spacing of feet reasonably. For linear motion, the arrangement of feet is determined by the positions of nodes. The node positions of axial and radial modes are roughly the same. Taking the axial mode as an example, its displacement (*w*) can be simply expressed as

where *K* is the vibration amplitude, *n* is the order, *θ* is the angle, with R1 direction being 0 degrees and counterclockwise direction being positive, *ω* is the angular frequency, *φ* is the phase.

The angular positions of the nodes (*θ_node_*) can be expressed as

$$\theta_{node}=\frac{(2k+1)\pi}{2n}$$

where *k* = 1, 2,,,2*n*-1.

When n = 3, *θ_node_* = 30°, 90°, 150°, 210°, 270°, 330°. When n = 4, *θ_node_* = 22.5°, 67.5°, 112.5°, 157.5°, 202.5°, 247.5°, 292.5°, 337.5°. When n = 5, *θ_node_* = 18°, 54°, 90°, 126°, 162°, 198°, 234°, 270°, 306°, 342°. The vertices of different polygons represent nodes of different order modes.

**Note S2.** Determination of key parameters.

The size and spacing of the feet mainly affect the modal frequencies and displacements of foot end, thereby exerting an influence on vibration coupling and motion efficiency. The determination of the parameters is primarily based on the degeneracy of the third-order axial modal frequencies and the displacement of foot end. Specifically, due to the asymmetrical structure of the configuration, the two third-order axial modal frequencies differ significantly, and the error needs to be adjusted to less than 3%. The displacement of driving foot is required to be greater than 1 μm to achieve effective motions. Key parameters are determined through simulation analysis.

The selection of the angle is the result of a comprehensive consideration of the degenerate frequencies of the third-order axial modes (for achieving high-efficiency rotational motions), the relative positions of the driving feet and nodes (for achieving high-efficiency linear motion), and motion stability. To determine the influence of the angle and foot length on the degeneracy of axial frequencies, modal analyses were conducted separately. The selection of the angle needs to comprehensively consider the size of the piezoelectric ceramics and the relative positions of foot 2 and 3 to the nodes. If the angle is set to 120°, the size of the axial piezoelectric ceramic must be reduced to 20°, making it difficult to excite the corresponding mode, and at this angle, foot 2 and 3 are far from the nodes. Therefore, the range of angles between the legs is set from 90° to 110°. The relationship between the angle and the frequency difference of the third-order axial modes is shown (see Fig. S2). As the angle increases, the axial frequency difference also increases. However, with a larger angle, the center of mass is closer to the center of the ring, resulting in more stable motion. At the same time, foot 2 and 3 are closer to the nodes, which has less influence on the motion of foot 1. Therefore, an angle of 110° is finally selected. At this angle, the error between the two third-order axial frequencies is 1.62%, which allows the excitation of traveling waves. Meanwhile, feet 2 and 3 are located close to the nodes, exerting a small influence on the linear motion of foot 1, which can achieve more efficient linear motion.

Simulations were performed by adjusting the foot length (see Fig. S2). As the foot length increases, the difference between the two axial modal frequencies also increases. To ensure the degeneracy of the axial modal frequencies, the foot length should not exceed 5 mm. The specific value needs to be further determined based on the displacement at the foot end. The relationship between foot length, displacement, and displacement difference was obtained through simulation (see Fig. S2). Considering both the degeneracy of the axial frequencies and the displacement at the foot end, a foot length of 4.6 mm was selected. The overall structural parameters of the robot are shown (see Fig. S3).

**Note S3.** The discussion about sampling frequency and pulse frequency.

Resolution refers to the minimum stable motion step size measured experimentally, while the motion step size is determined by the number of vibration cycles of the foot end. The number of vibration cycles (*n*) is governed by the frequency of the AC excitation signal (*f_e_*), duty cycle (*d_c_*), and pulse frequency (*f_p_*) (see Equation (3)). Pulse frequency refers to the frequency of the applied pulse signals. For instance, a frequency of 5 Hz means that there are 5 cycles of pulse signals per second. The duty cycle is defined as the ratio of the actual signal application time to the total period within each pulse signal cycle (see Fig. S8A). The frequency of the excitation signal was fixed at the optimal excitation frequency of 58.0 kHz, the minimum duty cycle of the power supply was set to 1%. We gradually adjusted the pulse frequency until the minimum stable step size was measured. As illustrated in Fig. R3, the step size at 4 Hz is 0.98 μm, and that at 5 Hz is 0.81 μm. Stable stepped displacement data could not be obtained at 6 Hz. Therefore, the result corresponding to 5 Hz (pulse frequency) represents the minimum stable step size, which is the resolution.

$$n=\frac{f_{e}d_{c}}{f_{p}}$$

The minimum sampling frequency configured by the software is 1 kHz. The data distribution characteristics under three sampling frequencies (1 kHz, 5 kHz, and 10 kHz) were compared. The collected data were subjected to low-pass filtering, and the stability of the filtered data was evaluated while ensuring that no signal distortion occurred. At a sampling frequency of 1 kHz, the data contained substantial noise, and fluctuations persisted even after filtering, resulting in poor data stability. In contrast, the filtered data at 5 kHz and 10 kHz exhibited relatively high stability. However, a higher sampling frequency leads to slower processing speed, thus 5 kHz was selected as the optimal sampling frequency.

**Note S4.** Calculation method for resolution of rotational motions.

Under continuous sinusoidal excitation, the robot operates in a macroscopic high-speed mode; under pulse signal excitation, the robot performs stepping motion with micrometer-level step size. The measurement methods for these two modes are shown (see Fig. S5). In the low-speed motion mode, a laser displacement sensor is used to measure the step size. After filtering the data with processing software, the corresponding step size is calculated. The minimum achievable step size of the robot is defined as its resolution, where the linear resolution can be directly obtained from the data, while the rotational resolution needs to be calculated using a formula.

In the high-speed motion mode, a high-definition camera is used to record the motion. A ruler grid plate is placed under the glass substrate, and key frames are processed using Adobe Premiere video editing software to calculate the speed.

The resolutions during rotational motions cannot be obtained directly from the data. For rotational motions in pulse mode, when the rotation angle is small (as shown in Fig. S5), the center of the robot may experience a certain offset during motion. Assuming the center shifts from O1 to O2, the schematic can be seen in Fig. S5:

$$\theta=\frac{x_{1}}{d_{1}}=\frac{x_{2}+x_{3}}{d_{2}+d_{3}}=\frac{x_{1}+x_{2}+x_{3}}{\text{L}-\text{L}\tan\frac{\theta}{2}}$$

$\theta\approx1-\sqrt{1-\frac{2(x_{1}+x_{2}+x_{3})}{\text{L}}}$ where *θ* is the angular resolution of the robot, *x*_1_​+*x*_2_​ and *x*_3_​ are the minimum step sizes measured by the two laser displacement sensors, and L is the length of the rectangle, and it is twice the distance from the laser measurement point to the center of the robot. Using Equation (4) and Equation (5), the rotational resolutions of the robot can be approximately obtained.

**Note S5.** The scheme and characteristics of wireless power supply.

The compact integrated power supply scheme is shown (see Fig. S6). The power supply uses a 3.7 V lithium battery to provide power to both the boost circuit and the ESP32 chip. The boost circuit raises the 3.7 V voltage to ±50 V [34]. The ESP32 outputs PWM signals through its pins, which are then used by the operational amplifier in the subsequent comparator output circuit. The comparator output circuit utilizes the voltage comparator within the operational amplifier, which is powered by a dual supply (the ±50 V generated by the boost circuit). Ultimately, the comparator produces excitation voltage signals with different frequencies and a value of 100 V_p-p_, thereby driving the robot’s motion.

Subsequently, a prototype PCB for the power supply was fabricated. The boost circuit prototype measures 30 mm × 30 mm and weighs approximately 3.2 g; the comparator output circuit prototype also measures 30 mm × 30 mm and weighs about 5.4 g. Output characteristic tests were conducted (see Fig. S6). The power supply successfully outputs excitation signals for controlling the robot’s linear and rotational motions, with frequencies of 58.0 kHz and 30.0 kHz, respectively. The two linear excitation signals have no phase difference, while the two rotational excitation signals have a 90° phase difference, meeting the design requirements.

**Note S6.** The relationship between load and resolution.

The load-resolution experiment has been performed to demonstrate the relationship between load and resolution. For wired motion, under low load conditions, as the load increases, the resolution decreases. The resolution is 0.68 μm under a load of 40 g. So an appropriate load can enhance resolution. The resolutions under heavy load conditions are also shown, the resolution is 0.83 μm under a load of 200 g, when the load reaches 400 g, the resolution can still reach 1.21 μm. The robot can maintain micron-level resolution even under heavy load conditions.

For wireless motion, under low load conditions, the robot can maintain high resolution, the resolution is 0.63 μm under no-load condition. The resolutions under heavy load conditions are shown, the resolution is 0.84 μm under a load of 100 g, when the load reaches 200 g, the resolution can still reach 1.28 μm. As a result, the robot can still maintain micron-level resolution even under heavy load conditions (see Fig. S11B-C).

**Supplementary Figures**


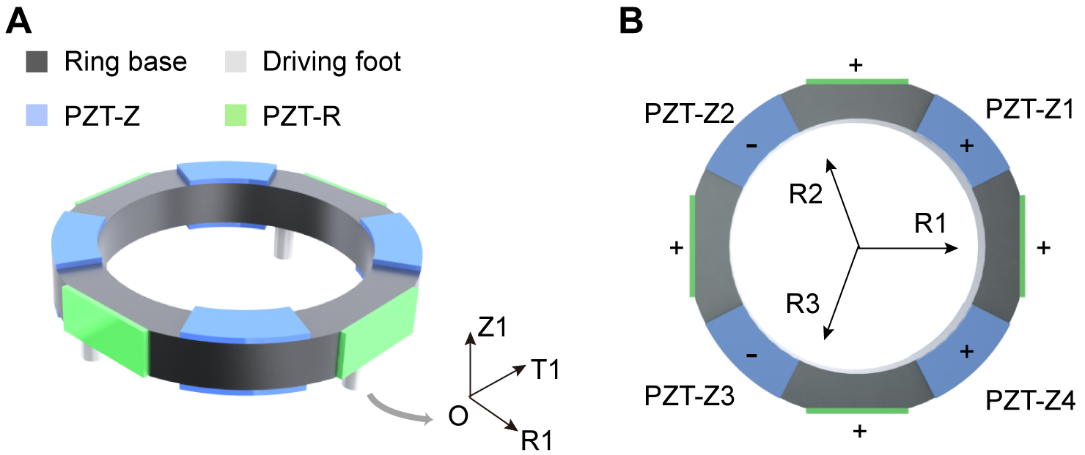


**Fig. S1.** The structure of robot and polarization directions of ceramics. (A) The structure of robot. robot only consists of one ring base, three driving feet, four radial piezoelectric ceramics (PZT-R), and eight axial piezoelectric ceramics (PZT-Z). For clarity, the driving feet are designated as foot 1 to foot 3, and a coordinate system O-RTZ is established at the tip of each foot, with the corresponding radial direction defined as the R direction, the Z direction oriented vertically upward, and the T direction determined according to the right-hand rule. Similarly, the radial ceramics at the corresponding foot positions are named PZT-R1 to PZT-R4, while the axial ceramics are divided into four groups, and are designated as PZT-Z1 to PZT-Z4. The naming and polarization methods of the ceramics are shown. Piezoelectric ceramics in different directions excite bending vibrations in corresponding directions. By utilizing different vibration modes, both linear and rotational motions can be achieved. (B) The polarization directions of ceramics. The axial ceramics PZT-Z1 and PZT-Z2 have the same polarization direction, while PZT-Z3 and PZT-Z4 have opposite polarization directions. And the radial ceramics on the opposite side have opposite polarization directions.


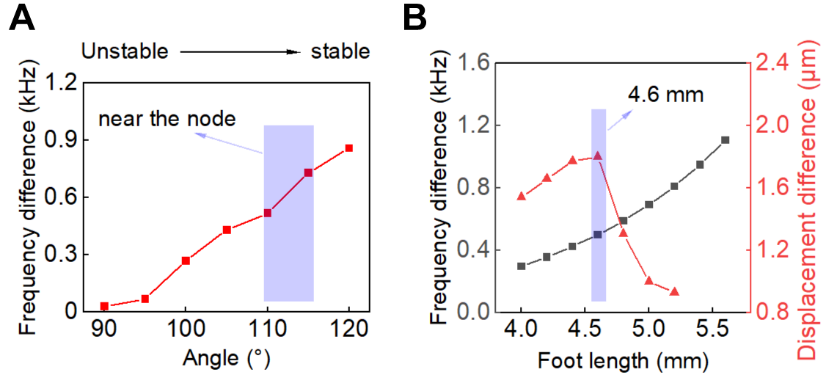


**Fig. S2.** Relationship between various parameters and frequency difference. (A) The relationship between angle and axial frequencies, as angle increases, the frequency difference increases, but the stability has improved. (B) The relationship between foot length and axial frequencies and displacement difference, as foot length increases, the frequency difference increases, and the displacement difference first increases and then decreases.


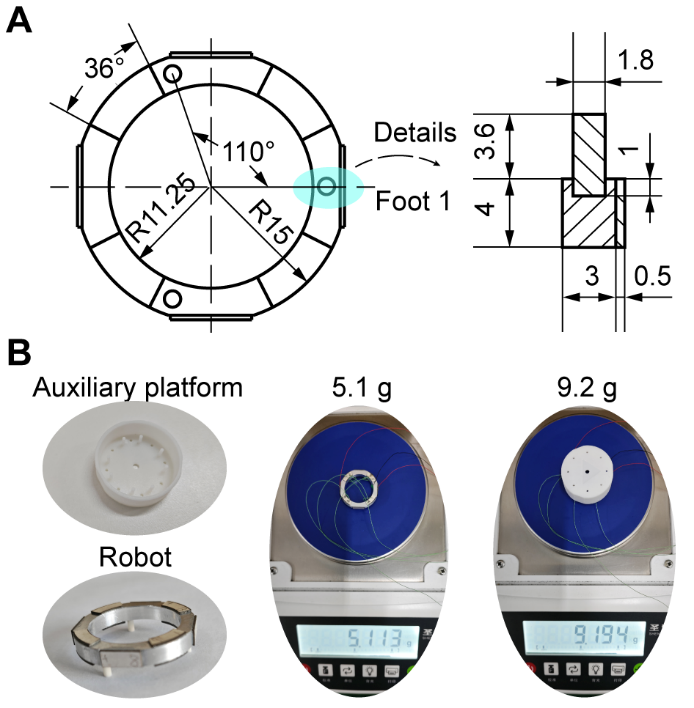


**Fig. S3.** The structural parameters and prototype of robot. (A) The structural parameters of robot. (B) The prototype of robot. The structural parameters were determined through simulation analysis. The inner and outer radius of the ring base are 11.25 mm and 15 mm, respectively. The thickness of the ring is 4 mm. All three feet have a diameter of 1.8mm and a length of 4.6 mm. For the convenience of loading, an auxiliary platform (3D printing) is placed on the robot. The size of the robot is 30 × 30 × 7.6 mm^3^, with a weight of 5.1 g. The overall size of the prototype is 38 × 38 × 15.5 mm^3^, with a weight of 9.2 g.


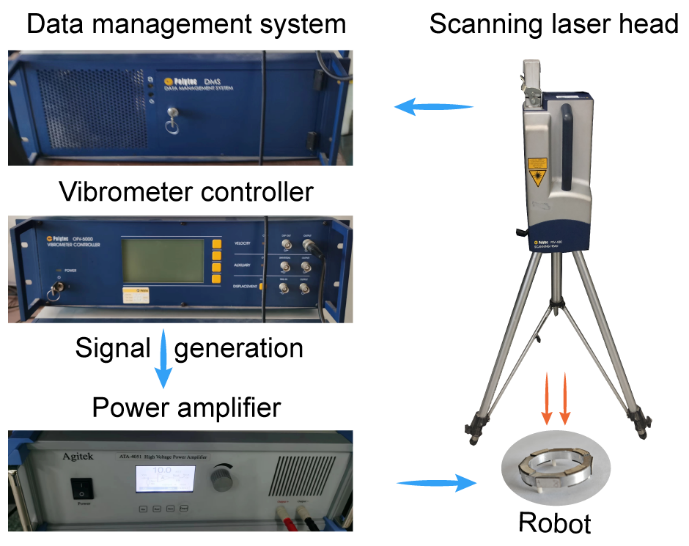


**Fig. S4.** Test method for vibration characteristics of robot. The instrument mainly includes a scanning laser head, a vibrometer controller, a power amplifier (ATA-4051, Aigtek Inc., China) and data management system. The vibrometer controller can generate sinusoidal signals from 0 to 1000 kHz, the power amplifier is used to amplify the voltage. The scanning laser head can generate a laser, which is vertical to the measured plane, and the tested data is stored in the data management system.


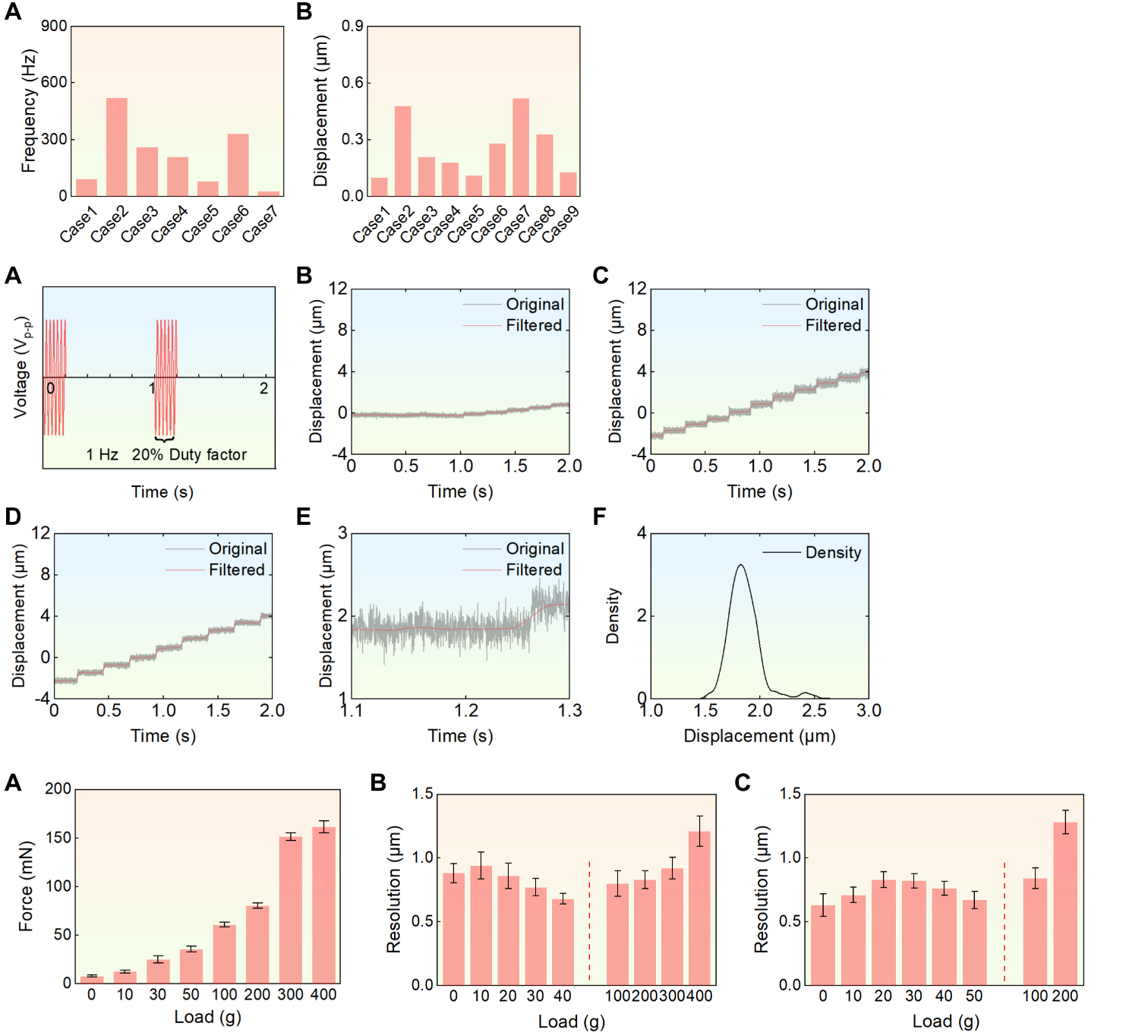


**Fig. S5.** The influence of different errors on frequency and displacement. (A) The influence of different errors on frequency. Where case 1 to case 4 refer to the influence of manufacturing errors, case 5 to case 6 refer to the influence of assembly errors, and case 7 accounts for the influence of boundary conditions. The following dimensional offset values represent the maximum limits of fabrication errors. Specifically, case 1 refers to a 0.1 mm elongation of the piezoelectric ceramics, case 2 corresponds to a 0.1 mm increase in the thickness of the ring, case 3 denotes a 0.04 mm widening of the ring, case 4 represents a 0.1 mm shortening of the feet, case 5 indicates a 0.1 mm positional offset of the piezoelectric ceramics, case 6 involves the influence of the adhesive layer (0.1 mm), and case 7 involves the influence of gravitational force. (B) The influence of different errors on displacement. Where case 1 to case 4 refer to the influence of manufacturing errors, case 5 to case 7 refer to the influence of assembly errors, case 8 accounts for the influence of boundary conditions, and case 9 considers the influence of the laser spot position. Specifically, case 1 to case 6 are the same as before, case 7 involves the influence of the 3D printing shell, case 8 refers to a modification of boundary condition from free condition to clamping condition, and case 9 means a 0.1 mm positional offset of the spot.


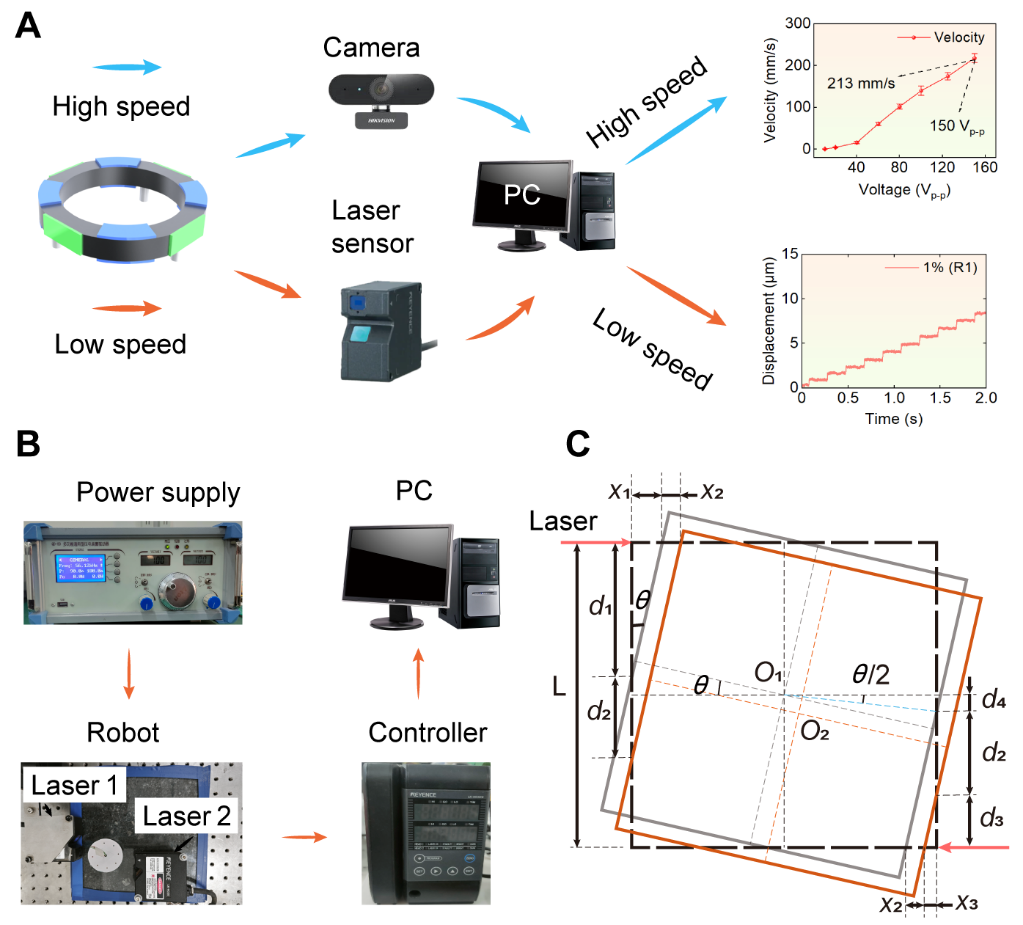


**Fig. S6.** Test methods for the motion characteristics of robot. (A) Measurement schemes for high-speed and low-speed motions. (B) Measurement methods for resolution characteristics. (C) Calculation method for resolution of rotational motions.


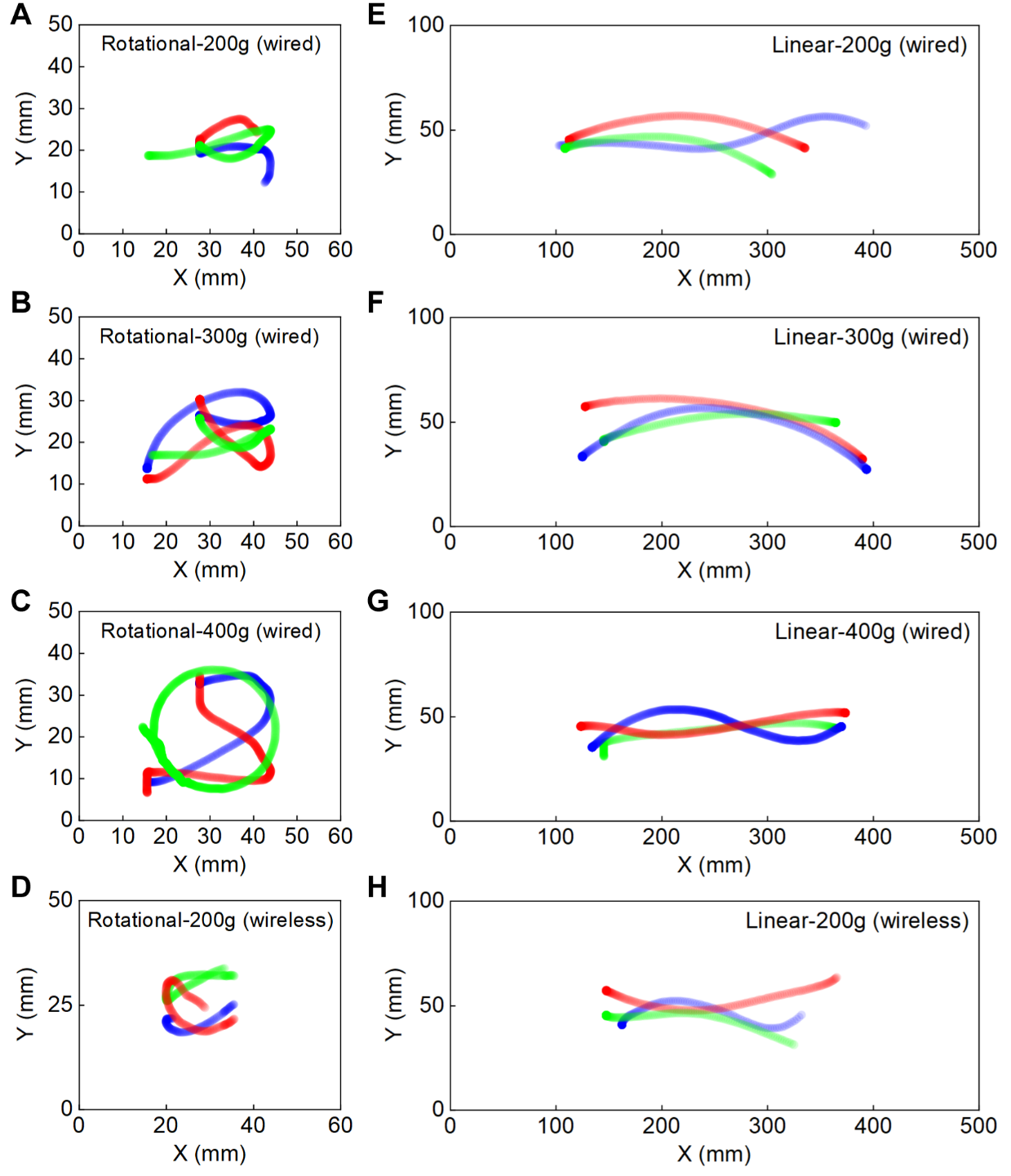


**Fig. S7.** Trajectory deviations of robot. (A) Trajectory deviations of rotational motions under a load of 200 g. (B) Trajectory deviations of rotational motions under a load of 300 g. (C) Trajectory deviations of rotational motions under a load of 400 g. (D) Trajectory deviations of untethered rotational motions under a load of 200 g. (E) Trajectory deviations of linear motion under a load of 200 g. (F) Trajectory deviations of linear motion under a load of 300 g. (G) Trajectory deviations of linear motion under a load of 400 g. (E) Trajectory deviations of untethered linear motion under a load of 200 g.


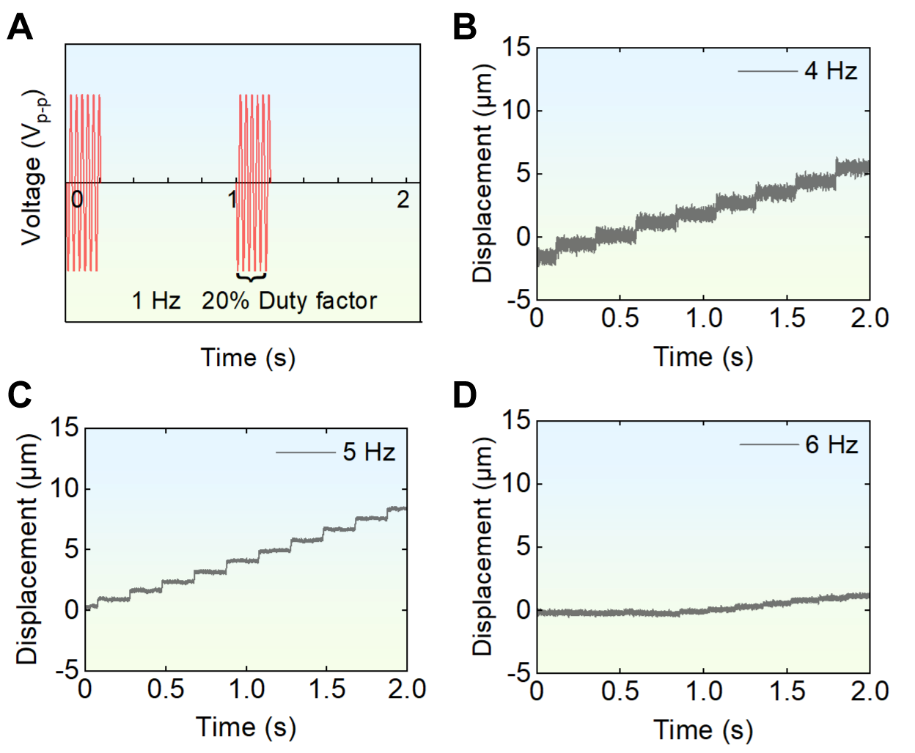


**Fig. S8.** The explanation of pulse signal and step displacement under different pulse frequencies. (A) The explanation of pulse signal. (B) Step displacement under 4 Hz pulse frequency. (C) Step displacement under 5 Hz pulse frequency. (D) Step displacement under 6 Hz pulse frequency.


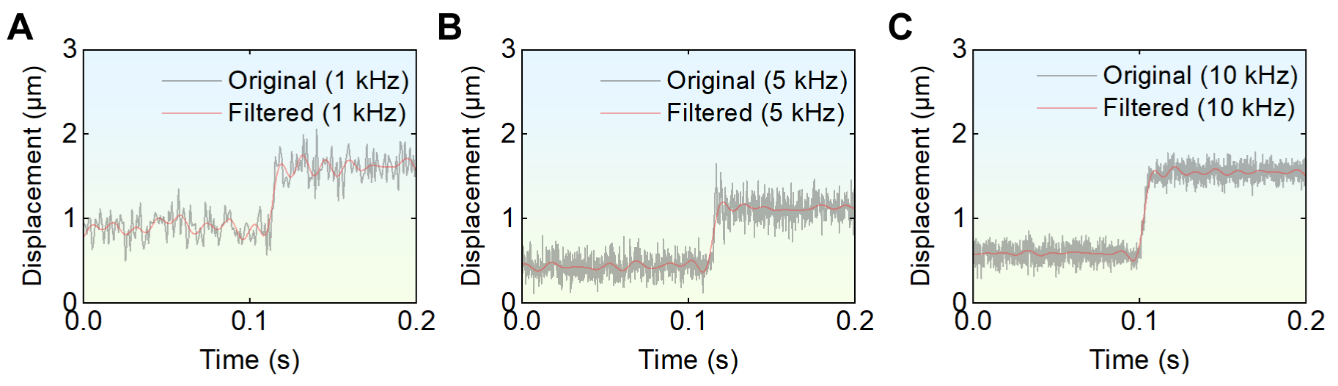


**Fig. S9.** The data under different sampling frequencies. (A) A sampling frequency of 1 kHz. (B) A sampling frequency of 5 kHz. (C) A sampling frequency of 10 kHz.

**
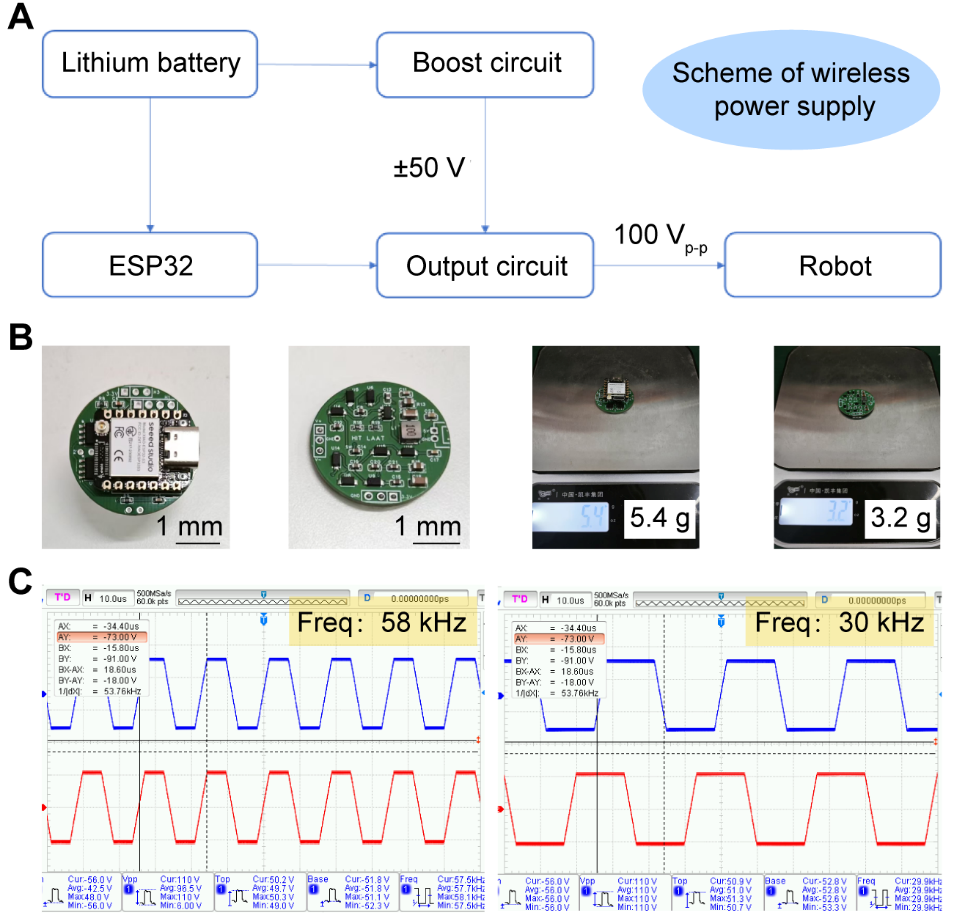
**

**Fig. S10.** The scheme, prototype and output characteristics of wireless power supply. (A) The scheme of wireless power supply. (B) The size and weight of wireless power supply, which contains two circuit boards. (C) The output characteristics of wireless power supply.


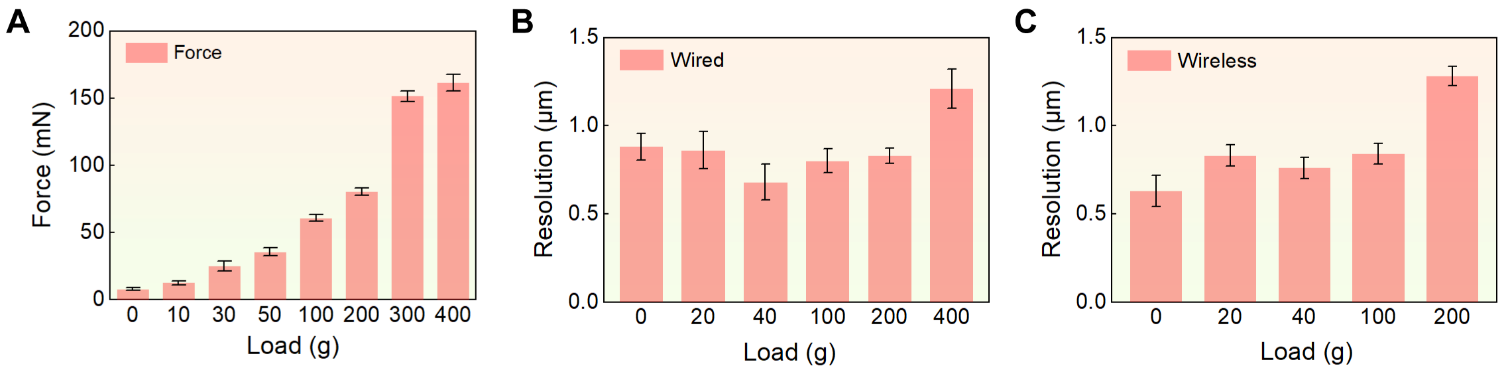


**Fig. S11.** The relationship between load and force, load and resolution. (A) The relationship between load and force. (B) The relationship between load and resolution in wired motion. (C) The relationship between load and resolution in wireless motion.


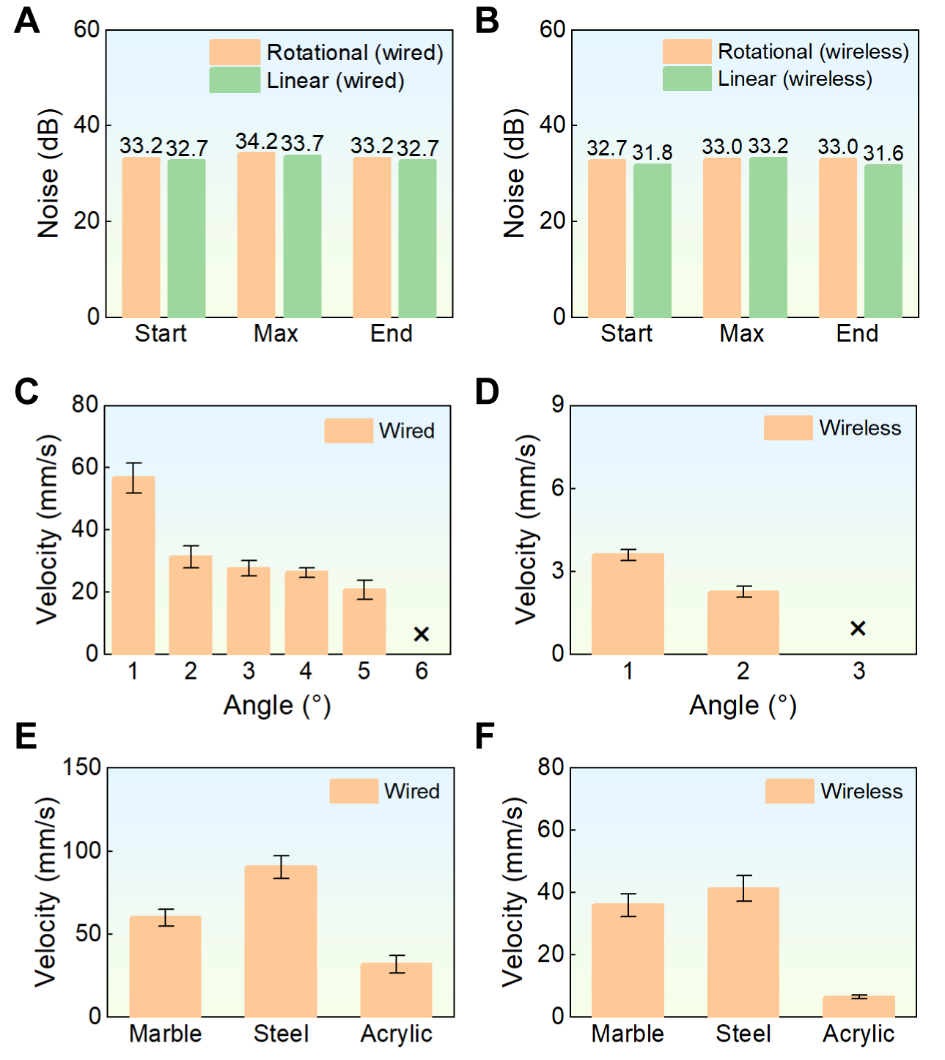


**Fig. S12.** The noise, climbing ability and adaptability of robot. (A) The noise of robot during wired motion. (B) The noise of robot during wireless motion. (C) The climbing ability of robot during wired motion. (D) The climbing ability of robot during wireless motion. (E) The moving surface adaptability of robot during wired motion. (F) The moving surface adaptability of robot during wireless motion.


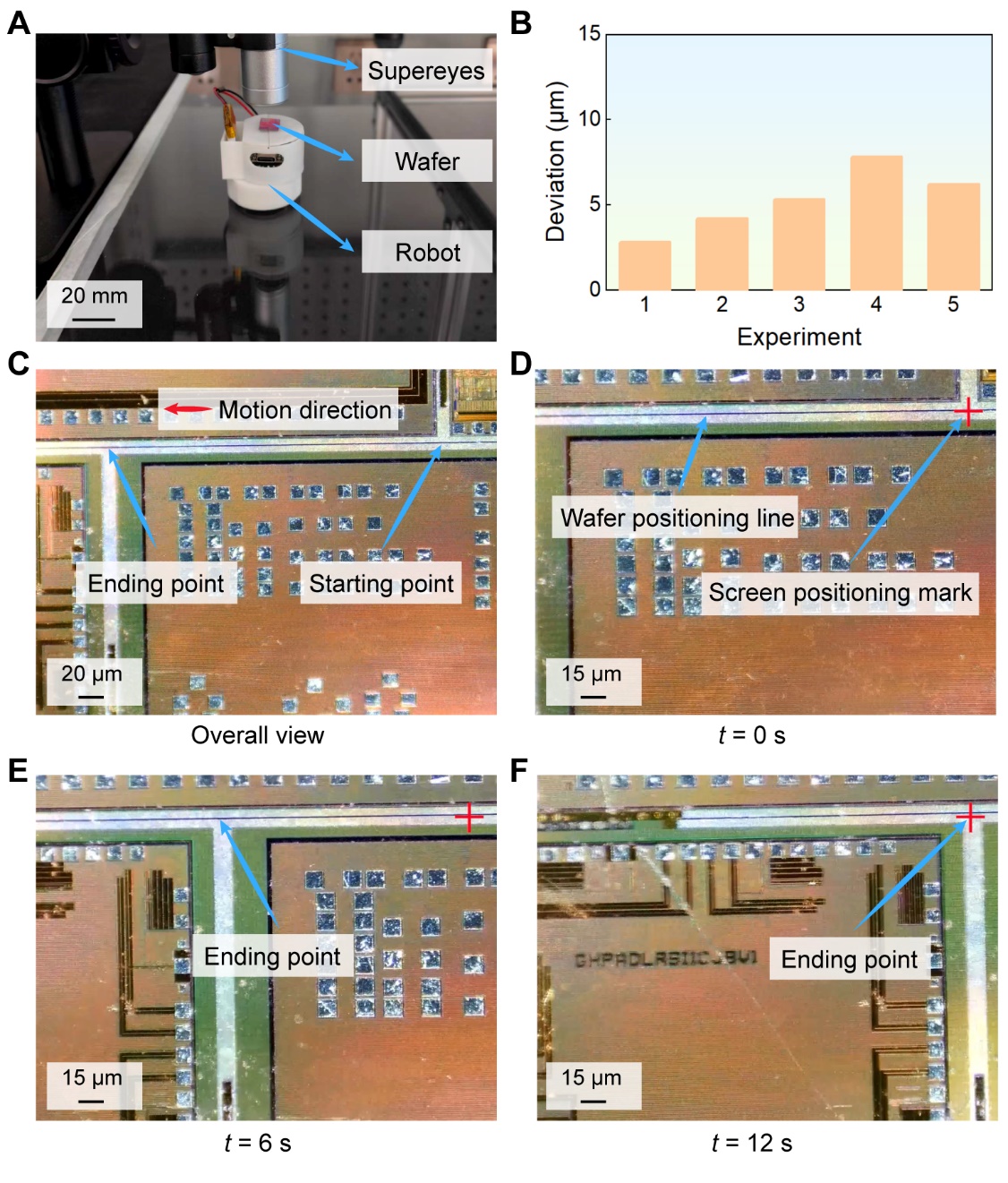


**Fig. S13.** Wafer inspection experiment. (A) The wafer inspection experiment. (B) Deviations of robot during multiple wafer inspection experiments. (C) The overall view of wafer detection area. (D) The image at the beginning moment. (E) The image at the midpoint. (F) The image at the ending moment.

**Supplementary Table**

**Table S1.** Influence of different numbers of feet on characteristics of ring-shaped robot.

| Number of feet | Stability | Load capacity | Over-positioning | Speed | Control | Choose |
| --- | --- | --- | --- | --- | --- | --- |
| 3 | Stable | Strong | No | Fast | Easy | √ |
| 4 | More stable | Stronger | Yes | Relatively slow | Relatively complex |  |
| 6 | More stable | Stronger | Yes | Relatively slow | Relatively complex |  |

**Table S2.** Comparison of simulation and experimental frequencies

| Frequency (kHz) | Third-order axial 1 | Third-order axial 2 | Fourth-order axial |
| --- | --- | --- | --- |
| Simulation | 30.0 | 30.5 | 56.2 |
| Experimental | 30.0 | 30.0 | 58.0 |
| Error | / | 1.67% | 3.10% |

**Table S3.** Comparison of simulation and experimental displacement

| Displacement (μm) | Linear motion:  D_R1_ | Rotational motion:  D_T1_ |
| --- | --- | --- |
| Simulation | 6.78 | 2.60 |
| Experimental | 5.82 | 3.04 |
| Error | 16.49% | 14.47% |

**Table S4.** Comparison of wired and wireless characteristics

| Characteristics | Wired | Wireless |
| --- | --- | --- |
| Linear speed(mm/s) | 213（150V_p-p_） | 77（100V_p-p_） |
|  | 136（100V_p-p_） |  |
| Rotational speed(°/s) | 548（150V_p-p_） | 318（100V_p-p_） |
|  | 394（100V_p-p_） |  |
| Load(g) | 400 | 200 |
| Resolution(μm) | 0.81 | 0.63 |
| Power consumption（W） | 2.01（100V_p-p_） | 1.75（100V_p-p_） |
| Flexibility | Strong | Stronger |

**Table S5.** Comparison of characteristics with other similar small robots.

|  | Our robot  Tethered/Untethered | Li et al. AQPR [40] | Hariri et al. [44] | Qu et al. [50] | Zhou et al. [54] | Goldberg et al. [55] |
| --- | --- | --- | --- | --- | --- | --- |
| Size (mm) | 38 | 30 | 90 | 21 | 55 | 45 |
| Weight (g) | 9.2/28.5 | 6.9 | 21 | 1.2 | 45 | 2.8 |
| Speed (mm/s) | 338/93 | 255 | 203.5 | 170 | 31.5 | 172 |
| CoT | 130.5/66.0 | 64.4 | 11.8 | 80.0 | 287.9 | 83.9 |

1. CoT can be calculated by Equation (6): while *P* is power consumption, *m* is the total weight of robot, *v* is the corresponding speed.

$\text{CoT = }\frac{P}{mgv}$ (6)
